# Coal Ash Triggers an Elevated Temperature Landfill Development: Lessons from the Bristol Virginia Solid Waste Landfill Neighboring Community

**DOI:** 10.3390/environments11090201

**Published:** 2024-09-14

**Authors:** Reagan Patton Witt, Marcelo I. Guzman

**Affiliations:** Department of Chemistry, University of Kentucky, Lexington, KY 40506, USA

**Keywords:** VOCs, landfill, public health, pollution, climate change, coal ash

## Abstract

Landfills for disposing of solid waste are designed, located, managed, and monitored facilities expected to comply with government regulations to prevent contamination of the surrounding environment. After the average life expectancy of a typical landfill (30 to 50 years), a large investment in the construction, operation, final closure, and 30-year monitoring of a new site is needed. In this case study, we provide a holistic explanation of the unexpected development of elevated temperature landfills (ETLFs), such as that in the city of Bristol (United States) on the border of the states of Virginia and Tennessee, including the initial role played by coal ash. Despite the increasing frequency of ETLF occurrence, there is limited knowledge available about their associated environmental problems. The study uses mixed (qualitative, quantitative, and mapping) methods to analyze (1) the levels of odoriferous reduced sulfur compounds, ammonia, and volatile organic compounds (VOCs) emitted, (2) the ratio of methane to carbon dioxide concentrations in five locations, which dropped from unity (normal landfill) to 0.565, (3) the location of gas well heads with gradients of elevated temperatures, and (4) the correlation of the filling rate (upward of ~12 m y^−1^) with depth for registered events depositing coal ash waste. The work identifies spatial patterns that support the conclusion that coal ash served as the initiator for an ETLF creation. The case of the city of Bristol constitutes an example of ETLFs with elevated temperatures above the regulatory United States Environmental Protection Agency (EPA) upper threshold (65 °C), having alongside low methane emissions, large production of leachate, land subsidence, and a large production of organic compounds. Such landfills suffer abnormal chemical reactions within the waste mass that reduce the life expectancy of the site. Residents in such communities suffer intolerable odors from fugitive emissions and poor air quality becomes prominent, affecting the well-being and economy of surrounding populations. Conclusive information available indicates that the Bristol landfill has been producing large amounts of leachate and hazardous gases under the high pressures and temperatures developed within the landfill. A lesson learned, which should be used to prevent this problem in the future, is that the early addition of coal ash into the landfill would have catalyzed the process of ETLF creation. The work considers the public health risks and socioeconomic problems of residents exposed to emissions from an ETLF and discusses the efforts needed to prevent further incidents in other locations.

## 1. Introduction

### Landfills for Waste Management.

Landfills for disposing of solid waste are designed, located, managed, and monitored facilities expected to comply with government regulations to prevent contamination of the surrounding environment. As of 2018, there are 1269 active municipal solid waste landfills operating in the United States [1], which not only aim to reduce the amount of waste transferred to the environment but also prevent disease transmission [2]. For example, the Bristol Virginia solid waste (BVSW) landfill opened in the city of Bristol, Virginia, in 1998 to dispose of mainly municipal waste during the next 30 to 50 years, but it occasionally received solid industrial waste [3]. The BVSW landfill was designed to reduce the operation costs of the two previous landfills that are now closed. The site selected for the BVSW landfill was an old abandoned quarry with limestone and dolomite bedrock [4]. The landfill quarry was initially coated by a 1.524 mm thick high-density polyethylene geomembrane and covered with a clay layer. For leachate and groundwater management, crushed stones were placed above the clay layer to create another permeable layer. The quarry side walls were also coated with another geomembrane layer held by wire mesh. A recent estimate of the landfill depth was 76–84 m, where 500 tons (out of 1600 tons allowed) of waste were disposed of daily under normal operation [3]. This landfill, as of September 2022, has been closed and waste collected from residents of Bristol is transported to the Eco-Safe landfill in Blountville (Tennessee) [5].

Landfills can have a large social impact on surrounding communities because their emissions into the air can be a public health threat [6]. Landfills also decrease the average value of adjacent land due to hazards, noise, pests, and water supply contamination [7]. A case of a city that was recently impacted by a landfill-associated problem is that of Bristol, whose citizens started to complain about odors (e.g., rotten eggs and chemical scents) around the BVSW site in December 2020 [3]. As time passed, residents perceived more pungent odors than before and started to report health problems that worsened at night and in clustered periods. The symptoms reported included nose bleeding, coughing, headaches, and difficulty breathing [8]. In the year 2021, the nonprofit organization Hope for Bristol was created by local residents, who began raising awareness while requiring authorities to solve the issue [9]. Eventually, on 25 April 2022, the matter was assessed as an elevated temperature landfill (ETLF), partially explaining the complaints of residents [3].

Before ETLF formation, municipal solid waste (MSW) landfills typically exhibit temperatures from 30 to 55 °C, but occasionally upper temperatures up to 65 °C can be reached due to anaerobic biodegradation [10]. Correlated concentrations of methane (45–60%) and carbon dioxide (40–60%) are expected at a ratio of CH_4_/CO_2_~1 under normal landfill conditions [11]. Approximately <3% N_2_, <1.5% O_2_, <1% H_2_, and <20 ppmv CO are found in a normal landfill before a temperature increase occurs [10–12], while subsurface combustion can be presumed when ~1000 ppmv CO is present [13]. Anaerobic biodegradation of landfill waste exerting <0.5 kPa pressure [11] is associated with the settlement of MSW landfills at a rate of 0.5–3%/year [10].

### Background on ETLFs.

ETLFs have been recently recognized as environmental problems but there is limited information to guide their identification [14]. A list of landfills experiencing elevated temperatures located in the United States, Israel, Greece, Norway, Japan, and Kuwait is provided in Table S1 (Supporting Information) [3,10,14–27]. This list with 20 entries corresponds to 39 locations as a study in Florida reported 20 cases. ETLFs can occur, exhibiting a wide range of temperatures and depths. Some known common characteristics of ETLFs are (1) an elevated temperature (T > 65 °C) [14], (2) a decreased methane-to-carbon dioxide molar ratio [10], (3) an increased molar ratio of carbon monoxide (>20 ppmv) and hydrogen (>2%) [10] gases, (4) leachate with a high biological oxygen demand (BOD), (5) a large production of leachate [10], (6) an observable subsidence of land (>3%/year), (7) a large production of gaseous organic compounds [28,29], and (8) an increase in gas pressure (>0.5 kPa) [10]. Because some biological and fermentation activity in landfills can potentially create temperatures >65 °C [30], the persistence of the eight indicators listed above is used to diagnose ETLF conditions. Some known potential factors that can initiate the process of transforming a landfill into an ETLF are the presence of aluminum waste and/or incinerator ash waste, events involving surface fires, spontaneous combustion, and to a minor extent, air intrusion [10,20,31–34]. Such components or processes can lead to unique chemical reactions that contribute to the heating of the landfill through a continuous mechanism.

During the continuous growth of an ETLF, substantial amounts of gases and leachate are produced and released into the environment. Gases are released through breaks within the landfills’ daily soil coverage and if not captured, can continue polluting the surrounding environment to an ETLF. Leachate and moisture can also be introduced through waste solidification activities, dehydration of MSW precipitation, ineffective surface water management, and groundwater infiltration due to a damaged liner [11]. The accumulation of leachate in MSW can lead to poor heat transfer, causing an increased risk of elevated temperatures to form. Typically, a leachate removal system with a pump transfers the landfill liquid waste generated into a waste treatment system, but for an ETLF, copious quantities of liquid can be hard to manage and have been known to increase by 5–7 times the amount relative to normal landfill conditions [11,35]. ETLFs can become highly problematic to their surrounding communities because as the temperature in the landfill is unexpectedly high, they emit strong unpleasant odors with low levels of methane while creating large amounts of hazardous leachate with elevated concentrations or organic compounds [3]. In addition, as an ETLF experiences a rapid settlement of waste, there are self-propagating reactions that keep generating excessive heat [3].

### The BVSW Landfill as a Case Study of an ETLF.

Although the BVSW landfill problem is somehow unique, it shares commonalities with other ETLFs. For example, many United States’ ETLFs odor issues from high landfill temperature decomposition of organic waste have affected the nearby communities. Similarly, the social impact on the surrounding community of the BVSW landfill problem is common to other ETLFs, which experienced decreased property values and public health problems. The BVSW landfill holds the eight common characteristics of ETLFs introduced earlier and that have been observed in other ETLFs in the United States [3]. There are gas hot wells with T > 65 °C [14], the ratio [CH_4_]/[CO_2_] ≪ 1 [10], the molar ratio of CO and H_2_ gases are >20 ppmv > 2% [10], respectively, (4) the leachate has a high BOD and is produced in copious amounts [10], land subsidence occurs at a fast rate > 3% year^−1^, there are large emissions of VOCs [28,29], and the pressure has increased to values > 0.5 kPa [10].

Potential factors to initiate the process of transforming the BVSW landfill into an ETLF have remained elusive and should be considered. It must be noted here that neither candidate events involving the presence of aluminum waste or incinerator ash waste had been reported, nor surface fires, spontaneous combustion, or air intrusion [10,20,31–34]. Thus, none of the previous causes could be used to explain the puzzling creation of an ETLF in this case study. Reaching a deeper understanding of the key factors and processes that contributed to the creation of the BVSW ETLF, a representative case in the United States can inform the stakeholders widely of such problems and their environmental impact.

The main research gaps for the BVSW ETLF are to identify the mechanism leading to elevated temperatures and its originating location in the landfill, to understand the corresponding subsurface chemical reactions, to determine the gas and leachate composition and their variation, and to register long-term monitoring data of temperature changes, emitted VOCs, and settling rates that will contribute to understanding the evolution of ETLFs over time. Thus, this work presents an analysis of key information for the BVSW landfill based on data from two government agencies, the EPA and the Virginia Department of Environmental Quality (VDEQ) [36,37].

The work hypothesizes that waste such as coal ash, aluminum dross, or incinerator ash deposited at an earlier time could have initiated the transformation of the BVSW landfill into an elevated temperature landfill (ETLF). The work evaluates this hypothesis by showing examples of the abnormal chemical reactions occurring within the waste mass. The data explain the diel cycling for fugitive emissions and intolerable odors that affected the well-being and economy of Bristol’s residents. A detailed explanation of the development of this type of ETLF is provided together with an assessment of the population affected and the most current engineering strategies to address such problems in a broad context. This work contributes to constraining the research gaps listed above by correlating the presence of coal ash (as an initiator) with position and depth in the landfill. To the best of our knowledge, this is the first case study of the BVSW ETLF available in the peer-reviewed literature. The information below should be considered by policymakers to create strategies to identify, remediate, and mainly prevent such environmental problems from occurring elsewhere in the future with appropriate landfill management procedures.

## 2. Methods

In this case study, we provide the first in-depth examination in the peer-reviewed literature of the direct impacts of the transformation of the BVSW landfill into an ETLF. The focus of the work is to provide a detailed contextual analysis of the data in three reports examining multiple pollution events under different environmental conditions. Specifically, the study uses mixed (qualitative, quantitative, and mapping) methods to analyze (1) the levels of odoriferous reduced sulfur compounds, ammonia, and volatile organic compounds (VOCs) emitted, (2) the ratio of methane to carbon dioxide concentrations in five locations of the landfill, (3) the location of gas well heads with gradients of elevated temperatures, and (4) the correlation of the filling rate with the depth for registered events depositing coal ash waste. The work identifies spatial patterns that support concluding that coal ash served as the initiator for an ETLF creation. Background information on the BVSW landfill is provided together with a deep analysis of three reports. Increased pollution levels are compared with baseline data, and the environmental implications are discussed to benefit other communities that could eventually suffer the same type of problem. The health risk assessment of exposed landfill workers to emitted VOCs is reported. The work establishes relationships among the pollution events and the presence of previously deposited coal ash as the key factor originating the ETLF, which in turn affected the health and well-being of the surrounding community. Guidance for how to proceed with such a problem is provided to the general reader. Public health officers or members of an environmental protection agency/commission can find the factors to be evaluated to determine if an ETLF has been created and to evaluate if the integrity of the landfill technologies has been compromised resulting in an environmental hazard. The research study also educates the stakeholders, both general readers and policymakers, with insightful knowledge to identify ETLFs and develop strategies to help prevent future developments in other landfills.

### Studies of the BVSW Landfill.

**Scheme 1.**
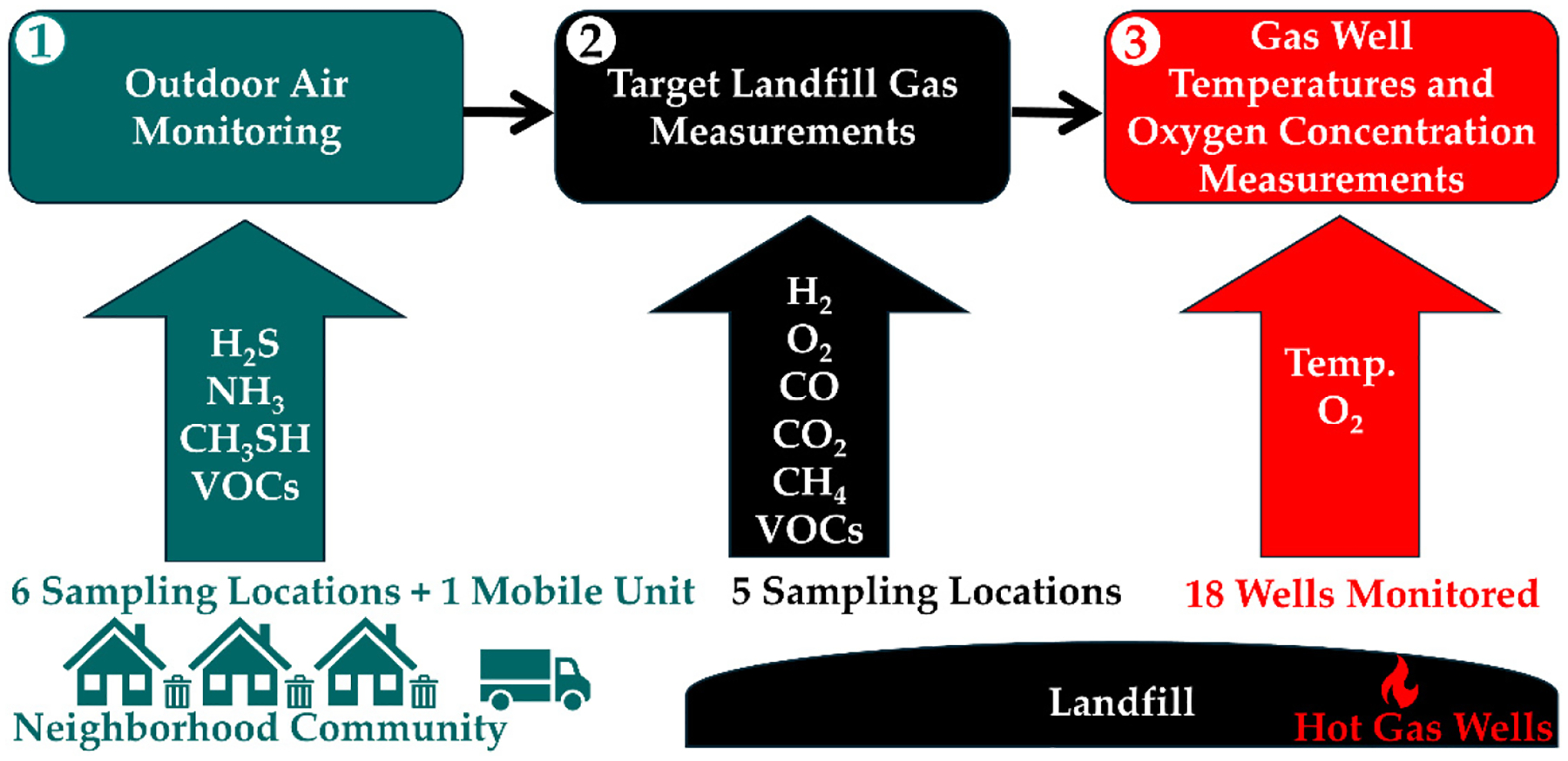
Representation of the various parameters reported from three studies.

Multiple pieces of work were conducted on the BVSW landfill, including (1) an outdoor air monitoring study [36], (2) targeted landfill gas measurements [37], and (3) a gas well temperatures and oxygen concentration monitoring study [38]. A panel review was also completed [3]. Overall, the work has reviewed the data in the reports [36–38] in three subsections; evaluated, filtered, applied grouping techniques, statistical analysis, comparisons to background/reference conditions, and developed new ways of visualization through reprocessing of data. By applying the methodology described herein, the research provides a deep examination of the information, identifying key findings to explain if coal ash, aluminum dross, or incinerator ash could have been key to initiating the transformation of the BVSW landfill into an ETLF while providing a new understanding of the scope of an ETLF. A diagram is provided in Scheme 1 with the various parameters reported below to facilitate a quick grasp of the material covered.

The datasets used in this work include measurements of air contaminants in the surrounding neighborhood [36] and inside [37] the BVSW landfill, which are publicly available through the EPA and VDEQ, respectively. Data considered irrelevant, based on exclusion criteria applied, are not discussed in this work. In more detail, data from these studies were excluded for analytes flagged in the reports because pollutants were not detected (ND) at the quantitation limit (QL) or reporting limit, below the QL, or below the minimum detection limit (MDL), exceeding the calibration curve range, exceeding the acceptable recovery range for the methods, or exceeding acceptable limits for the relative or absolute difference between duplicate samples. Other excluded data correspond to the evaluation of an instrument being inaccurate, offline, or near a facility with interferences such as a painting shop. After applying the exclusion criteria, the datasets from continuous monitoring devices were reviewed, and the species of interest were identified to peak during nighttime and vanish during daytime. Thus, the peaks registered in the nighttime data for odoriferous species of interest are reported here. Appropriate analytical procedures for sampling, zeroing, calibration of instruments, and selection of sampling locations were followed [36,37].

### Outdoor Air Monitoring.

A study conducted on the surroundings of the BVSW landfill under a contract with EPA sampled six stationary locations (three residential, one public utility, and two commercial) and multiple mobile units throughout the city of Bristol in both Tennessee and Virginia from 9 June to 22 July of 2021 [36]. Air analyses targeted the detection of VOCs (in locations 1 through 6), ammonia (NH_3_) (in location 1), hydrogen sulfide (H_2_S) (in locations 1, 2, 3, 4, and 6), and methyl mercaptan (CH_3_SH) through mobile monitoring. The six sampling locations and parameters monitored were selected to satisfy the goal of monitoring air in areas that had been linked to odor complaints to determine any association with hazardous substances and their concentrations [36]. The selection of these six locations was agreed between the EPA, VDEQ, the cities of Bristol, Virginia, and Bristol, Tennessee, and the Agency for Toxic Substances and Disease Registry (ATSDR) [36]. Other considerations that influenced the selection included equipment security; potential sources in the area; power availability; potential for human exposure; topography; and geographical coverage. The data from the surrounding neighborhood to the BVSW landfill were collected by Tetra Tech under a superfund technical assessment and response team (START) contract with EPA [36]. Data were measured with a Single-Point Monitor Flex for hydrogen sulfide (1 ppb–10 ppm range, 1 ppb resolution) and ammonia (10 ppb–150 ppm range, 10 ppb resolution), and a MultiRAE Pro for methyl mercaptan (0–10 ppm range, 100 ppb resolution) [36] are included in the data presented below in the outdoor air monitoring section. In addition, an AreaRAE with a photoionization detector was used for measurements of volatile organic compounds (VOCs, 0 ppb–2000 ppm, 1 ppb resolution) [36]. Additional samples collected with Summa canisters by the VDEQ showed the concentration of individual species < 10 ppb and were considered insignificant. A meteorological station (Davis Vantage Pro 2) was used to measure localized weather conditions (temperature, barometric pressure, wind direction and speed, precipitation, and relative humidity) [36].

### Targeted Landfill Gas Measurements.

Data from the BVSW landfill correspond to measurements completed on 16 November 2021 by Trinity Consultants sampling five locations detailed in the Results and Discussion section [37]. A figure including data measured by EPA method TO-15 for VOCs and EPA method 3C for atmospheric gases hydrogen, nitrogen, oxygen, carbon monoxide, carbon dioxide, and methane is included in the section on targeted landfill gas measurements [37]. The reported data below within the section targeted landfill measurements also include a figure that grouped the VOCs with similar properties for the case of (1) Freon 11 and 12, (2) hexane and heptane, (3) acetone and 2-butanone, (4) ethanol and isopropanol, and (5) benzene, toluene ethylbenzene, and xylenes (BTEX) together, while other compounds registered are not depicted due to their lower concentrations. Although this dataset also included analysis by other methods for formaldehyde, hydrogen sulfide, and methyl mercaptan among other species, their presence at low levels (below relevant detection) served as a criterion to exclude such information in this work. For simplicity, all chemical species written in chemical reactions are defined as gases unless their physical state is differently indicated.

### Gas Well Temperatures and Oxygen Concentration Measurements.

Additional temperature and O_2_ concentration data were collected from 18 different gas well heads from the northern and southern portions of the BVSW landfill that were obtained from public reports [3,38]. While these data cover the period from 8 August 2021 to 15 February 2022, the information from 14 December 2021 is excluded from our analysis due to a reported vacuum pump failure.

### Health Risk Assessment (*HRA*).

An *HRA* of workers at the BVSW landfill is performed for the VOCs detected. The *HRA* considers risk by inhalation of VOCs during a time shift (D) of 4 h spent outdoors for monitoring, inspections, machinery operation, waste management, waste disposal, and other needed activities at the five landfill locations studied [39]. The weekly exposure (E) is calculated with Equation (1) from their concentration in air ($C_{\text{air}}$) and the dimensionless exposure ranking (ER) obtained with Equation (2) [39]:

(1) $$E=\frac{F\times D\times C_{air}}{W}$$

(2) $$ER=\frac{E}{OEL}$$

where F=14 is the number of workers in attendance at the BVSW landfill [5], $C_{\text{air}}$ is the exposure amount (mg m^−3^), and W=40h is the duration of a working week. In Equation (2), $\text{OEL}(\text{mg}\;{\text{m}}^{-3})$ defines the occupational exposure limit determined through the time-weighted averages from reference values [40]. The range of ER values obtained are ranked in five categories referred to as exposure levels (EL): EL1 for ER<0.1,EL2 for 0.1≤ER≤0.5,EL3 for 0.51≤ER≤0.99,EL4 for 1≤ER≤2, and EL5 for ER > 2 [39].

The risk level (RL) of each VOC is obtained with Equation (3) [39]:

(3) $$RL=\sqrt{HR\times EL}$$

where HR is the hazard rate, a number from 1 (low HR) to 5 (high HR) based on reference values [40–44].

Following the previous analysis, the chronic daily intake of inhalation ($CDI_{\text{inhal.}}$) for each VOC is calculated with Equation (4) [39,45]

(4) $$CDI_{\text{inhal.}}\left(\frac{mg}{\text{kg day}}\right)=\frac{C_{air}\times IR\times ET\times EF\times ED}{BW\times AT}$$

to evaluate the health risk of exposed workers. In Equation (4), $IR=0.35\;{\text{m}}^{3}\;{\text{h}}^{-1}$ is the inhalation rate per hour [46], $ET=4\;\text{h}\;{\text{day}}^{-1}$ is the exposure time due to labor time spent in the sites, $EF=260\;{\text{days year}}^{-1}$ is the exposure frequency for a year of labor, ED=6 years is the exposure duration (accounting from 2016, when an increase in $C_{air}$ for benzene was recorded [3], until 2022, when the landfill was closed [5]), BW=70kg is the average body weight [46], and AT=10,950 days (≡30 years) is the average number of days a worker spanning a 50 year career lives after exposure [39].

The hazard quotient for inhalation of the VOCs ($HQ_{\text{inhal.}}$) provides a non-cancerous risk assessment based on Equation (5) [39,47]

(5) $$HQ_{\text{inhal.}}=\frac{CDI_{\text{inhal.}}}{RfD_{\text{inhal}}.}$$

where $RfD_{\text{inhal}}$. is the daily reference pollutant dose (mg kg^−1^ day^−1^) for human inhalation, which values are available (or calculated from the reference concentrations) in the literature [42,48]. A calculated $HQ_{\text{inhal.}}<1$ indicates there is not risk of a non-cancer hazard for the individual VOC. When HQ>1, there is a non-cancer health hazard risk to the workers [39,49]. The inhalation hazard index ($HI_{\text{inhal.}}$), which is the sum of total hazard quotient values computed for each VOC (at each location), is obtained as indicated in Equation (6) [50]

(6) $$HI_{\text{inhal.}}=\Sigma HQ_{\text{inhal.}}$$

## 3. Results and Discussion

### Findings of the Outdoor Air Monitoring.

**Figure 1.**
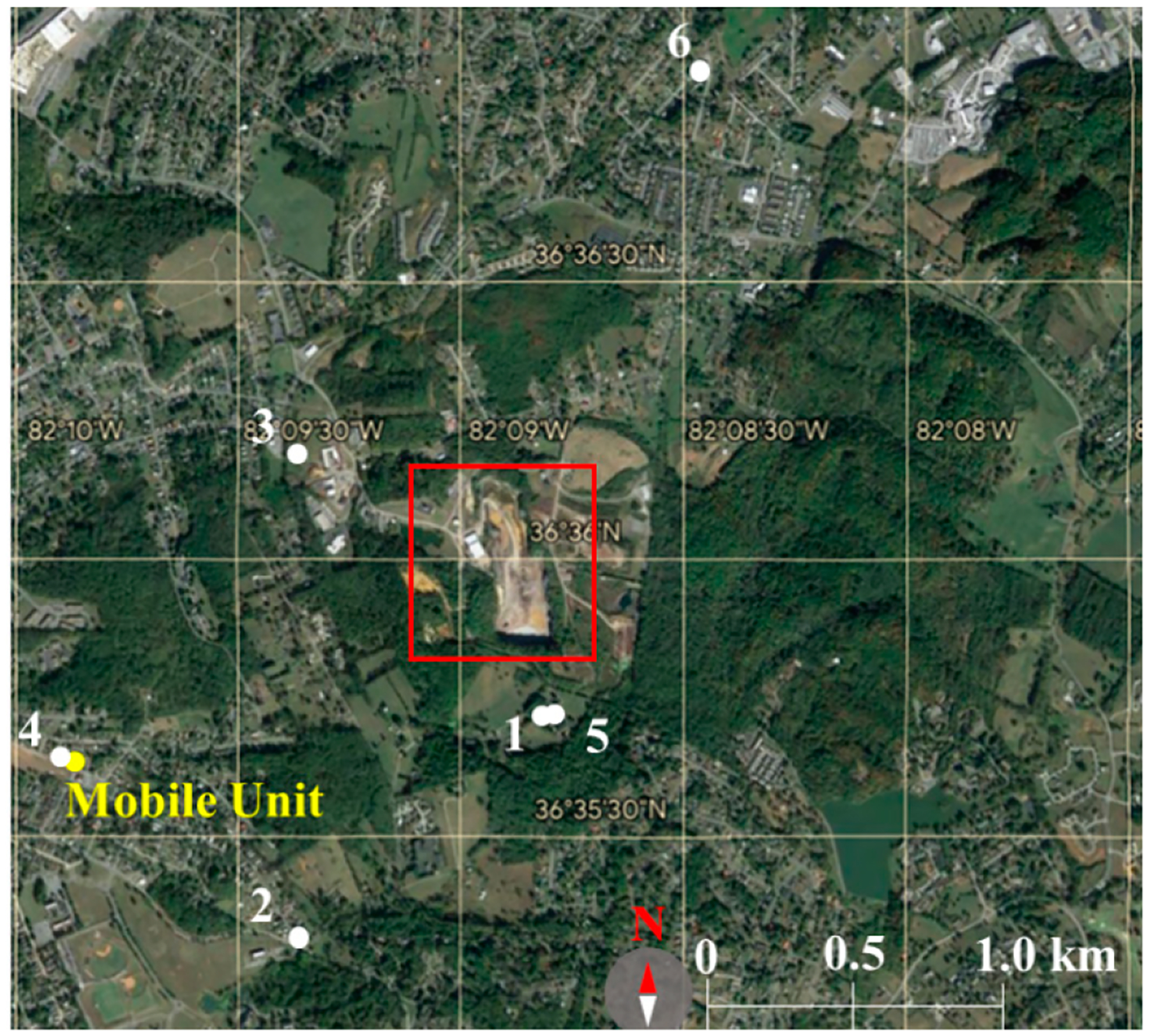
Six stationary locations (1–6) and a mobile unit sampled around the Bristol Virginia solid waste (BVSW) landfill marked in a red square, the address of is 2655 Valley Drive, Bristol Virginia, United States.

Figure 1 displays the six stationary sampling locations around the landfill [36]. Air analyses targeted the detection of VOCs (in locations 1 through 6), ammonia (NH_3_) (in location 1), hydrogen sulfide (H_2_S) (in locations 1, 2, 3, 4, and 6), and methyl mercaptan (CH_3_SH) through mobile monitoring. Nighttime measurements exceeding minimum odor detection thresholds for methyl mercaptan (1.6 × 10^−3^ ppm) [51], hydrogen sulfide (5.0 × 10^−4^ ppm) [52], and ammonia (4.0 × 10^−2^ ppm) [53] explain that neighbors around the landfill were affected by emissions.

**Figure 2.**
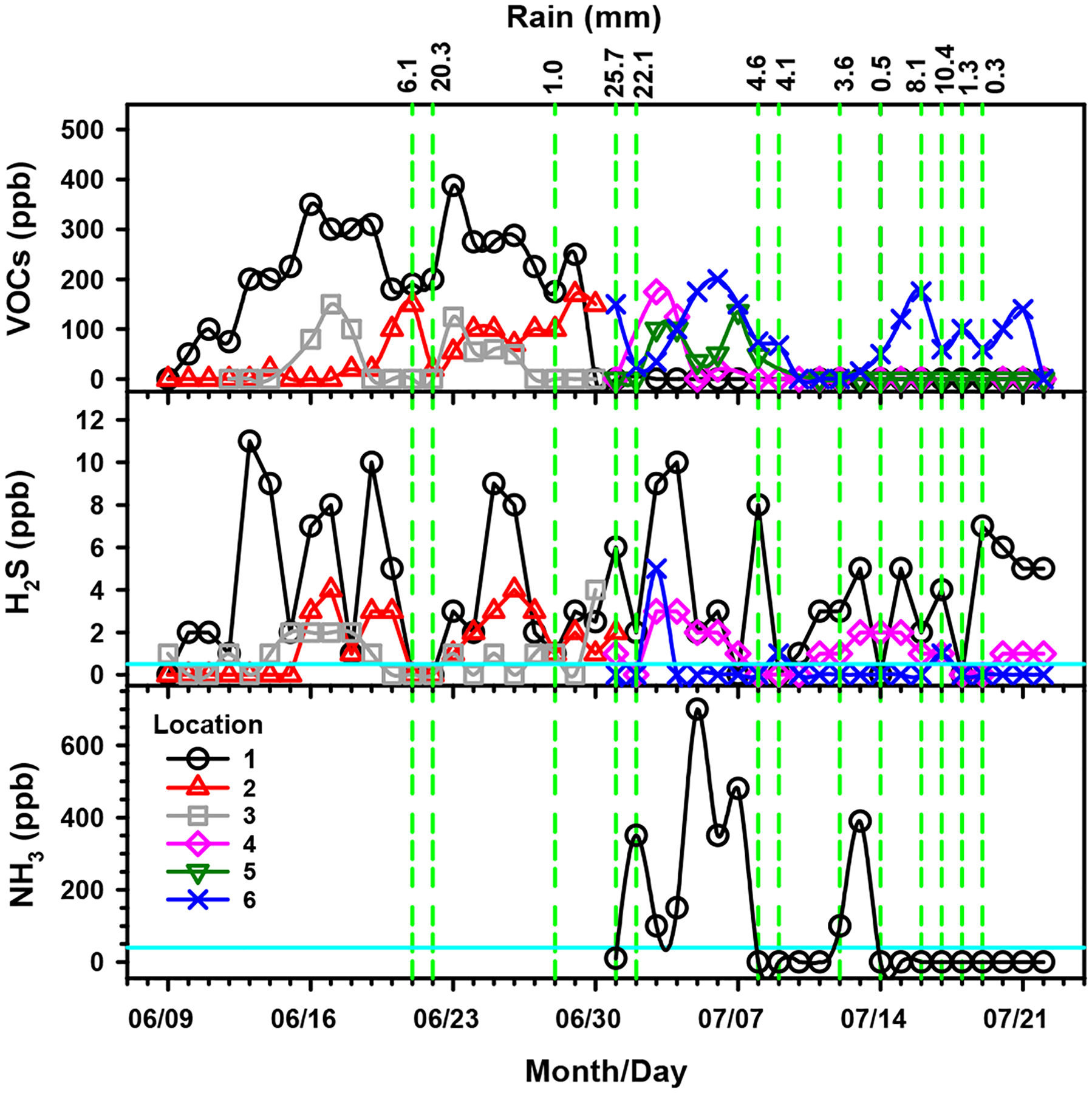
Average molar ratios at nighttime for volatile organic compounds (VOCs), hydrogen sulfide (H_2_S), and ammonia (NH_3_) for stationary monitoring around the BVSW landfill neighborhood (locations 1–6 in Figure 1) from 9 June to 22 July of 2021 (based on data from ref. [36]). The green dashed vertical lines correspond to rain events and the cyan horizontal lines mark the odor detection threshold for H_2_S and NH_3_.

The air monitoring study showed that the emission of VOCs increased at nighttime starting at 8:00 p.m. and eventually reached stable levels after midnight, which occasionally included maxima spikes about 2:00 a.m. The maxima nighttime concentrations for hydrogen sulfide and ammonia are presented in Figure 2. Stable maxima nighttime values for VOCs are presented in Figure 2. As explained above, VOCs were determined with a photoionization detector. The EPA contractor only reported valuable VOC measurements for nights when readings were stable. Precipitation causes a partial washout effect as rainfall scavenges volatile pollutants from the air. For example, after the rain on June 21, the decreased concentration of H_2_S falls below the odor detection threshold. However, abundant precipitation events such as those from July 1 and 2 that total 47.8 mm must also contribute to runoff spreading of pollutants that emit odoriferous species as evident from the spikes of H_2_S on July 3 and 4 for locations 1, 4, and 6 as well as NH_3_ on July 5 for location 1.

For comparison to the stationary monitoring data in Figure 2, mobile monitoring measurements in the surrounding urban area showed low VOC levels from 0 to 120 ppb (with averages ≤ 50 ppb). However, on two occasions, VOC levels reached 16 and 22 ppm on July 1 near the intersection of East Mary Street and Delaney Street and 13 ppm on 8 July near the intersection of Booher Road and Booher Springs. Simultaneously to the 13-ppm reading for VOCs, 0.1 ppm methyl mercaptan was confirmed (above the odor detection limit) during mobile monitoring at the same location [36].

### Findings of the Targeted Landfill Gas Measurements.

**Figure 3.**
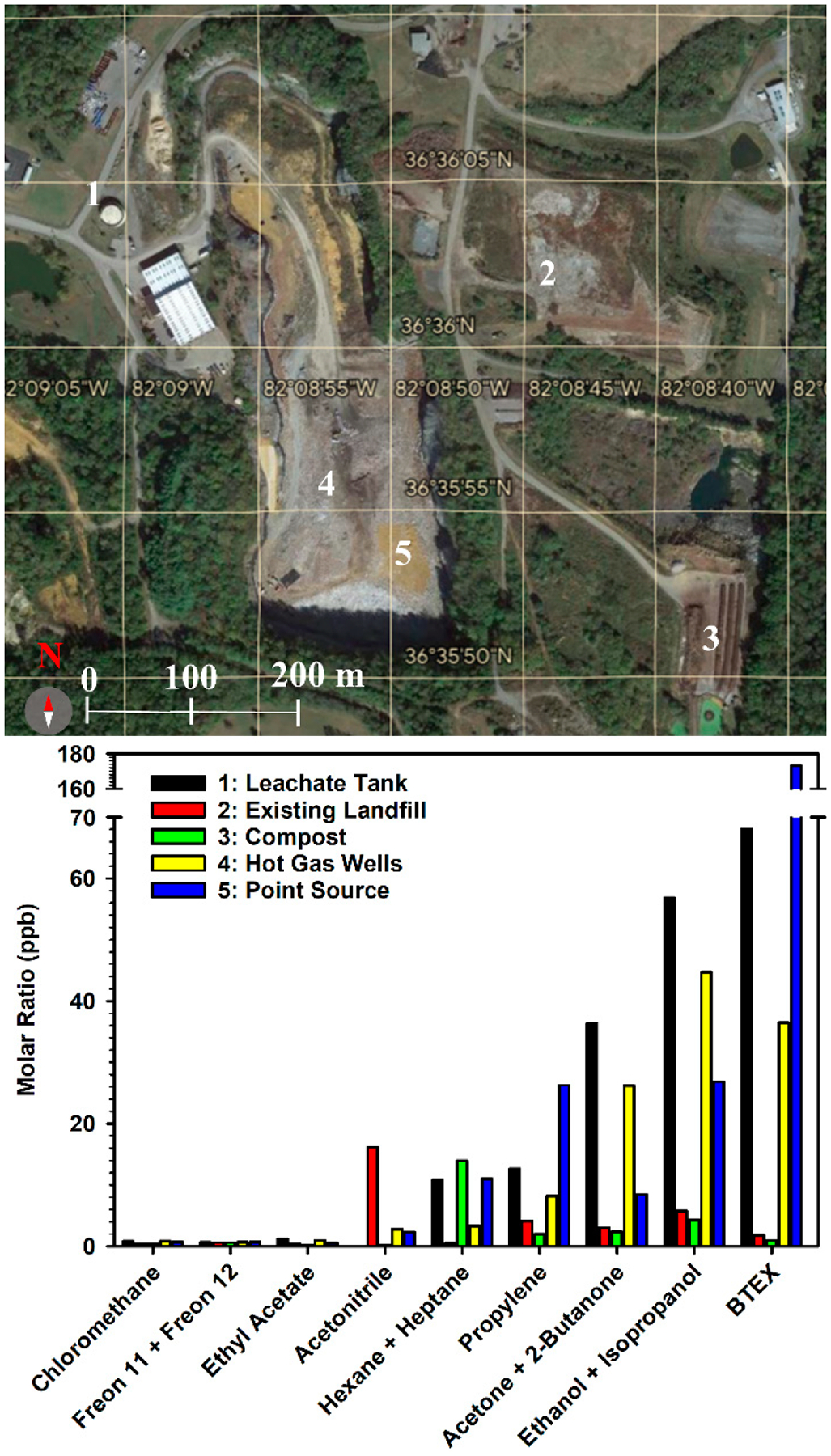
(**Top**) Five locations of the BVSW landfill sampled: (1) leachate tank, (2) the existing landfill, (3) compost, (4) hot gas wells, and (5) the point source. The map is a close-up of the red square featured in Figure 2. (**Bottom**) Bar chart for the molar ratio of organic species type in each location (based on data from ref. [37]).

This study was contracted by VDEQ and sampled five separate locations within the perimeter of the BVSW landfill to try to identify the source of emitted pollution to air: (1) the leachate tank, (2) the existing landfill, (3) the compost section, (4) the hot gas wells, and (5) gaseous emission point source where the release of gases through daily cover breaks proceeds. The existing landfill mentioned refers to the previously closed landfill. Measurements correspond to a 30-min sampling time on 16 November 2021 at these locations for analysis of VOCs, sulfides, formaldehyde, polycyclic aromatic hydrocarbons (PAHs), and atmospheric gases. The analysis of air pollutants sampled in the BVSW landfill leachate shows notably high concentrations of organic species (Figure 3). The largest accumulation of these organic species was found at the point source (location 5 in Figure 3) and the leachate tank (location 1) and minimal concentrations were found at the compost (location 3) and the existing landfill (location 2). The previous variation with sampling location can be explained as due to the major emission originating at the point source (location 5), close to the highest subsurface temperature, with transport through the leachate to produce the second-ranked emission at the leachate tank (location 1). The third-ranked emission (on its path from location 5 to location 1) proceeds from the gas wells (location 4). In contrast, due to their farther separation from the heated point source, the existing landfill (location 3) and the compost (location 2) show lower emissions. The aromatic hydrocarbons benzene, toluene, ethylbenzene, and xylenes (BTEX) are grouped in Figure 3, as well as the ketones (acetone and 2-butanone), the Freons (11 and 12), the acyclic hydrocarbons (hexane and heptane), and the alcohols (ethanol and isopropanol). The source of BTEX detected in the gas phase could not be related to solvent waste disposal. BTEX represents the column with the highest concentration across all sampling locations in Figure 3, with a large percentage being benzene [3].

**Figure 4.**
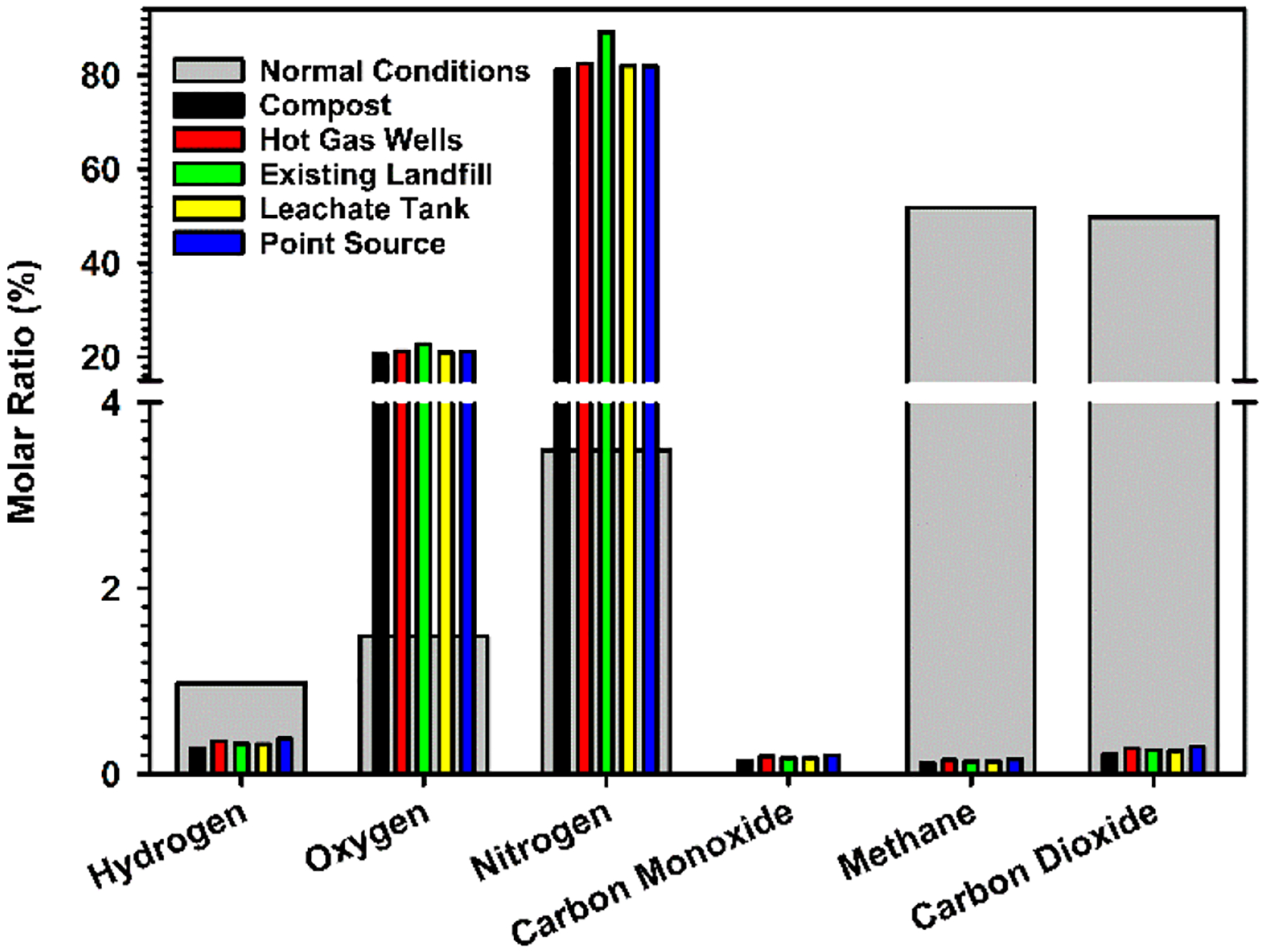
Comparison of the production of gases in the BVSW landfill under (wide gray bar) normal landfill conditions [10,13] and at the following locations of the BVSW landfill (adapted from ref. [37]) in Figure 3: (green bar) existing landfill, (black bar) compost, (red bar) hot gas wells, (yellow bar) leachate tank, and (blue bar) the point source. Underground gas piping systems interconnect the BVSW landfill property such as locations 2, 4, and 5 in Figure 3 [54,55].

The percent molar ratios of atmospheric gases sampled during this study are compared to those of normal landfill conditions [10,13] in Figure 4. Although the concentrations of hydrogen, carbon monoxide, methane, and carbon dioxide are minimal, they are still represented in Figure 4 to provide a comparison to the rather large values expected under normal landfill conditions. The normal conditions of this landfill display high levels of methane and carbon dioxide in Figure 4, which are expected for natural anaerobic biodegradation dominating under the surface layers. For comparison to the normal control values, a shift toward the large oxygen and nitrogen levels in the atmosphere was registered in the five locations of this study. The low concentrations of methane and carbon dioxide registered in the five locations of the BVSW landfill fall below unity, indicating an expected behavior from an ETLF. In consequence, there is a considerable drop in the production of methane and carbon dioxide (e.g., that would be expected from anaerobic biodegradation) as compared to the normal conditions and an increment of the heat and leachate released.

Although the N_2_ and O_2_ levels may look close to those in background air in Figure 4, the data cannot be interpreted as sampling atmospheric concentrations because CO, CO_2_, and CH_4_ are 8 × 10^3^–1 × 10^4^, 6–7, and 700–900 times higher than in a pristine environment, respectively. In other words, the gases emitted from the landfill are sampled as they have mixed with the surrounding air. The present data cannot support air intrusion as a plausible mechanism for ETLF development. The fact that the ratio [CH_4_]/[CO_2_] = 0.565 (±0.002) and is significantly smaller than 1 (value for a normal landfill) for all five locations, suggests that multiple sites in the landfill property are impacted by the elevated temperatures. A further discussion relating the measurement of landfill gas emissions to areas experiencing elevated temperatures is provided below.

### Findings of the Gas Well Temperatures and Oxygen Concentration Measurements.

**Figure 5.**
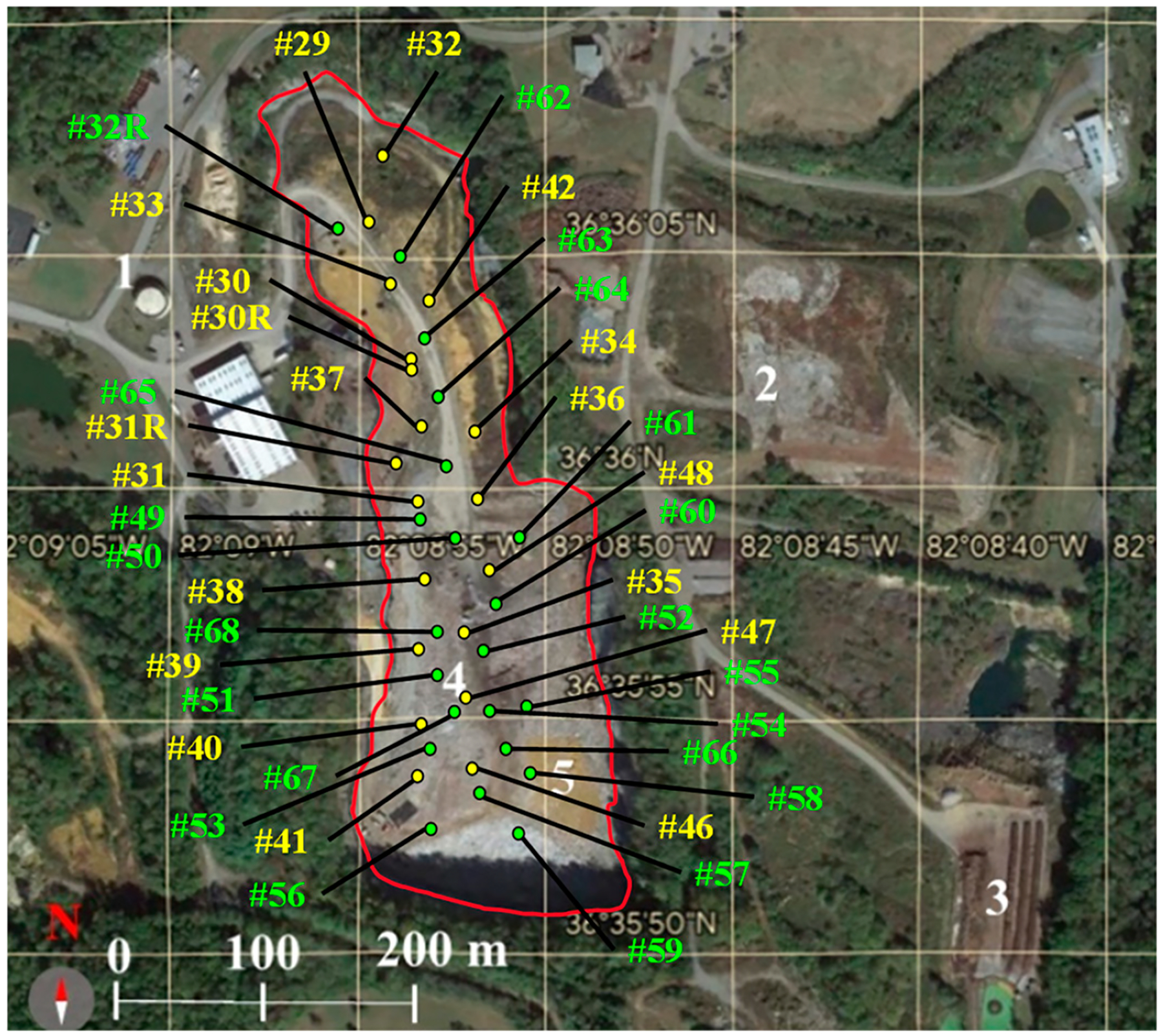
Close-up of the map in the red square featured in Figure 2 showing inside the quarry wall (red) of the landfill with the numbers for older (yellow, existing in 2016) and newer (green, existing in 2021) gas well heads (yellow). The five locations (1–5) in white font were defined in Figure 3.

Available gas wellhead temperatures [3] are reported in Table 1 for the gas well locations in Figure 5. As designated by the EPA, standard operating temperatures for landfills occur below 65 °C [14] unless specific and rare methanogenesis is present [30]. Similarly, the industry standard for oxygen concentration in interior gas wells is 2% for normal operating conditions [3]. The highest temperature occurs at gas well heads #66 and #67 in the southern portion and #64 and #65 in the northern portion, reaching 70 °C, as indicated in Table 1 [3]. Considering wells #64, #65, #66, and #67 are above the threshold of elevated temperature (65 °C) and that they exhibit high oxygen content (>2%), it is possible to suggest that both southern and northern portions are driving elevated temperature reactions while separated by regions of lower temperatures.

**Figure 6.**
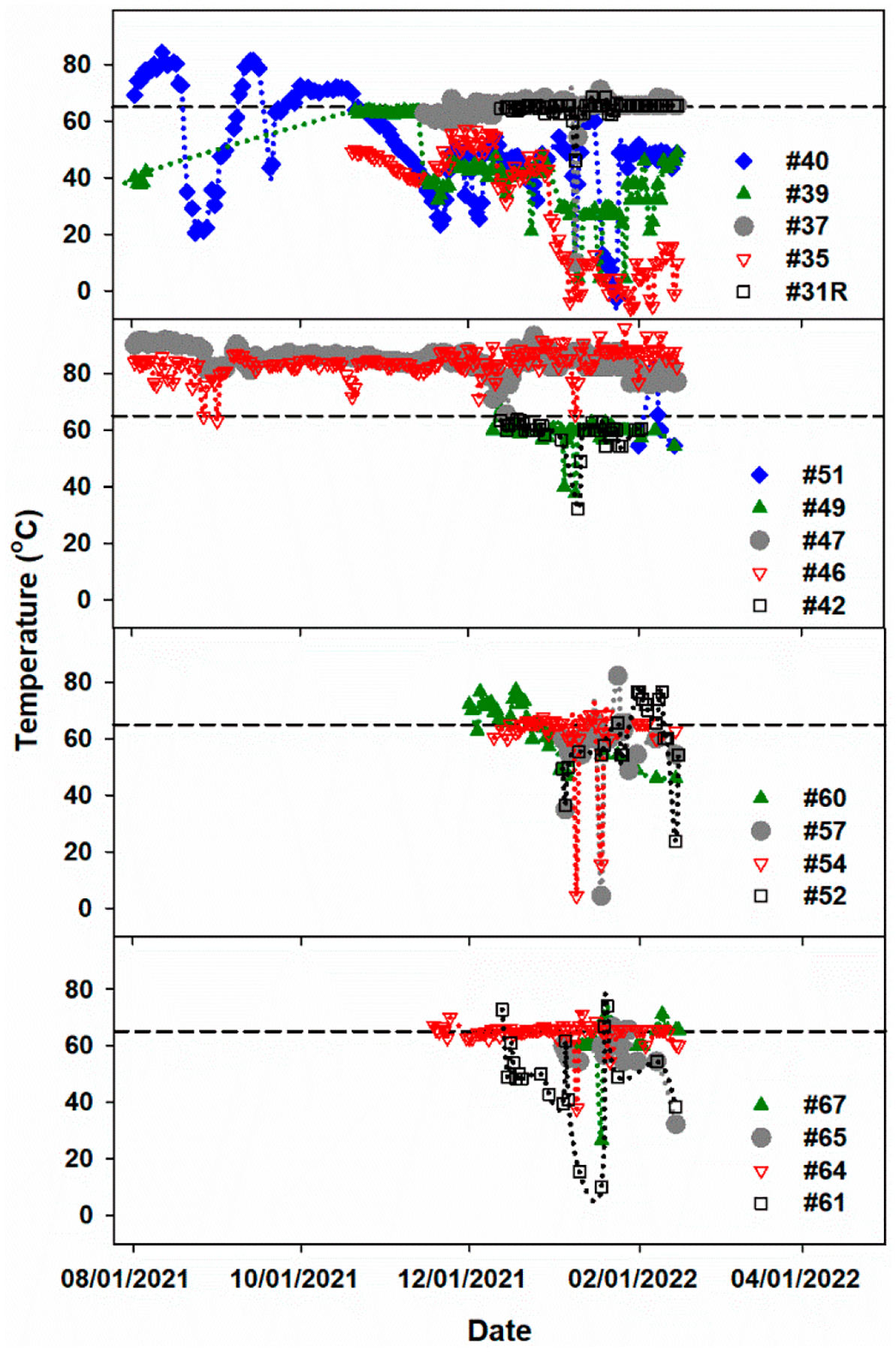
Temperature measurements of 18 different gas well heads based on locations represented in Figure 5. EPA’s operating upper-temperature limit of (dashed line) 65 °C for landfills [38]. The dotted lines connecting the measurements represent a guide to the eye only.

Additional elevated temperature data in Figure 6 were collected from 18 different gas well heads over 7 months. Remarkably, gas well heads #46 and #47 have high temperatures continuously averaging 84 and 85 °C, respectively [38]. Weather conditions (e.g., warmer weather and cooling rain) and operational activities (e.g., the addition of new waste, compaction, and soil coverage) can affect the daily temperature readings. However, as observed in Figure 6, the variation in temperature of gas well heads 46 and 47 is insignificant through time, a circumstance that can only be interpreted as a confirmation that these wells reached elevated temperatures from subsurface processes. High-temperature gas wells #66, #67, #46, and #47 are clustered gas wells located near the center of the southern portion of the landfill, which could indicate an ETLF source point. Larger fluctuations in temperature over time seem apparent in gas wells #35 and #39. Both wells # 35 and #39 are sandwiched between the ETLF zones of highest temperatures.

### Transformation of the BVSW Landfill into an ETLF.

Before exploring the cause of ETLF creation further, it is important to highlight the normal background conditions under which the BVSW landfill should have operated. MSW landfills include two different reactions under normal operating conditions; anaerobic biodegradation and aerobic biodegradation [3]. Anaerobic biodegradation by reaction R1 (exemplified for a hexose monosaccharide as the unit of biomass) dominates the degradation of solid waste < 65 °C with a ratio of methane to carbon dioxide that can exceed the ideal stoichiometry of this reaction, [CH_4_]/[CO_2_] ≥ 1 [3]:

(R1) $${\text{C}}_{6}{\text{H}}_{12}{\text{O}}_{6}(s)\to 3\;{\text{CO}}_{2}+3\;{\text{CH}}_{4}$$

**Figure 7.**
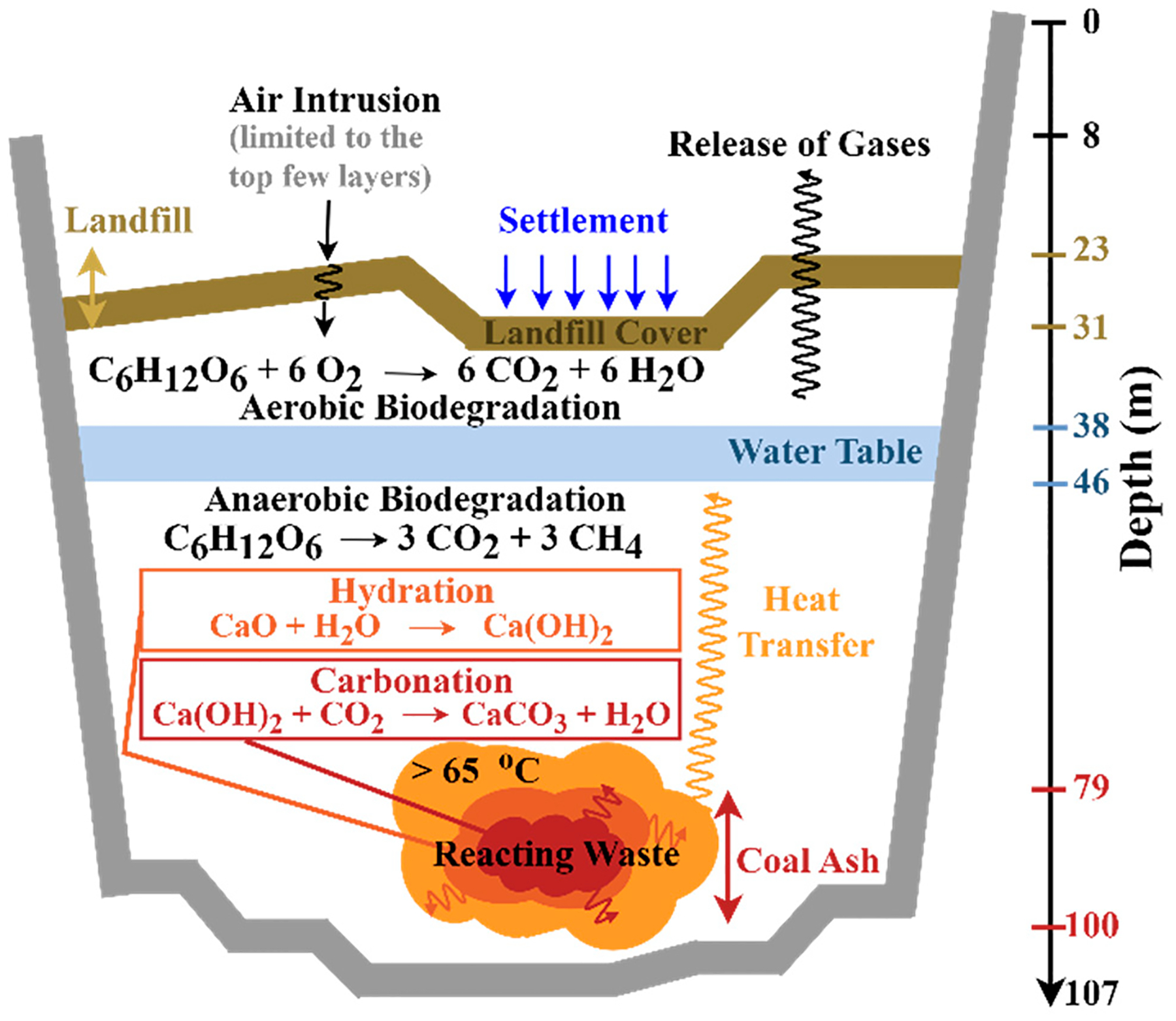
Aerobic reactions take place in the first few layers of the landfill. Waste at deeper levels is decomposed through anaerobic biodegradation at <65 °C with an enthalpy change $\Delta \text{H}=-145\;\text{kJ}\;{\text{mol}}^{-1}$. Air intrusion can cause surface portions of the landfill to participate in aerobic biodegradation with $\Delta \text{H}=-2815\;\text{kJ}\;{\text{mol}}^{-1}$, releasing large amounts of heat. With the addition of coal ash into the landfill, hydration $\left(\Delta \text{H}=-1164\;\text{kJ}\;{\text{kg}}^{-1}\right)$ and carbonation $\left(\Delta \text{H}=-1719\;\text{kJ}\;{\text{kg}}^{-1}\right)$ reactions are likely to proceed in the presence of CO_2_ generated in anaerobic reactions. The heat released through these reactions is transferred outward from the point source unless it is inhibited through highly saturated landfill masses or compaction. The gases produced through these reactions are released through breaks in the landfill cover. The depths provided are referred to a local ground level of 0 m at the highest landfill altitude.

Additionally, aerobic biodegradation reaction R2 occurs when oxygen is available, producing CO_2_ and water in the top layer

(R2) $${\text{C}}_{6}{\text{H}}_{12}{\text{O}}_{6}(s)+6\;{\text{O}}_{2}\to 6\;{\text{CO}}_{2}+6\;{\text{H}}_{2}\text{O}(l)$$

and releasing a significant amount of heat. Comparatively, aerobic reaction R2 creates −2815 kJ mol^−1^ of energy, while anaerobic reaction R1 only releases 5.2% (−145 kJ mol^−1^) of the previous heat [56]. Aerobic reactions are frequently responsible for landfill surface fires [3], proceeding at the cost of reducing the contribution of anaerobic reactions while increasing surface landfill temperatures. Figure 7 displays a summary of processes, including anaerobic and aerobic biodegradation that release heat, occurring in the BVSW landfill.

It has been proposed within the literature that the landfill’s waste layers can experience a shift from the production of methane by reaction R1 to a higher formation of carbon dioxide and water by reaction R2 with the intrusion of air. This minor pathway bringing O_2_ into the top few layers of the BVSW landfill results in a small contribution of water to the gas wells relative to infiltration due to precipitation. In theory, air intrusion could contribute to ETLF formation, creating elevated temperatures (>65 °C) after aerobic biodegradation [30]. However, air intrusion should result in a decreasing ratio of CH_4_ to CO_2_ ([CH_4_]/[CO_2_] < 1) and a temperature (T) in the range 55 ≤ T < 65 °C due to gas and temperature fronts resulting from reaction R2. The smoldering effect (associated with reaction R3)

(R3) $${\text{C}}_{6}{\text{H}}_{12}{\text{O}}_{6}\left(s\right)+5.7\;{\text{O}}_{2}\to 5.4\;{\text{CO}}_{2}+0.6\;\text{CO}+6\;{\text{H}}_{2}\text{O}(l)$$

is designated through high molar ratios of CO > 1500 ppm agreeing with the observations in Figure 4, waste temperatures T ≥ 65 °C, and settlement of the waste. However, an expected drop in the CH_4_ to CO_2_ ratio due to smoldering to [CH_4_]/[CO_2_] < 0.2 is not observed for the Bristol data in Figure 4. The sign of smoldering in an ETLF indicates the onset of thermal degradation of the waste, where incomplete combustion occurs due to limited oxygen reaching this area. In consequence, degradation byproducts such as odoriferous reduced sulfur compounds and VOCs are released into the atmosphere [10,35]. Finally, the absence of extremely high temperatures (700–800 °C) expected from combustion and pyrolysis proceeding by reaction R4 [57,58] discards the possibility of such processes in the BVSW landfill.

(R4) $${\text{C}}_{\text{x}}{\text{H}}_{\text{y}}(s)+{\text{O}}_{2}\to {\text{CO}}_{2}+{\text{H}}_{2}\text{O}$$

A discussion of an ETLF formation is provided next by considering the ETLF zones described above with predominant reactions R2 and R3 and a tetrahedron fire theory.

Based on the tetrahedron fire theory, combustion requires four components in the landfill: (1) a fuel source (the waste), (2) an oxidizer (oxygen from air intrusion), (3) an energy source (heat released by an exothermic reaction such as aerobic biodegradation), and (4) a chain reaction [35,59,60]. In the absence of other limitations, this chain reaction only stops when the fuel is depleted, oxygen is exhausted, or energy is absorbed into another source [59,60]. Events resulting from air intrusion are only likely to occur in the top few layers of the landfill and are not associated with deep elevated temperatures. Because the waste in the BVSW landfill is highly saturated and the high leachate levels of 0.2–0.5 million gallons per day in 2021, it is unlikely that air intrusion played a significant role in forming an ETLF in Bristol. Therefore, the tetrahedron fire theory and air intrusion are unsustainable theories to explain the BVSW landfill problem.

Among the nearly dozen known ETLFs in North America [3,14,15,19,20,61,62], there are several prominent cases to consider the initiating conditions toward the development of the ETLF at the BVSW landfill that are listed in Table S1 (Supporting Information). Table S1 also indicates that five other ETLFs (Bridgeton near Saint Louis, Hillside near Chicago [16], Countrywide near Akron [3,14], Middle Point near Nashville [14,18], and Rumpke near Cincinnati) [14,19] were formed in quarry landfills like the Bristol site. Furthermore, Table S1 (Supporting Information) points out the multiple U.S. locations that experienced the addition of coal ash, aluminum waste, or incinerator waste as a determining factor to increase the reactivity of waste in contact with liquid (leachate) [32]. Similar to the BVSW landfill, a few gas well heads of the Bridgeton landfill [10,14] experienced elevated temperatures > 90 °C and levels of CH_4_ that were low (~1000 ppm) and CO that were high (>1500 ppmv). The previous information contributed not only to the diagnosis of an ETLF at Bridgeton but also to map the point source of this heat to the southern quarry and a depth of ~98 m similar to the 89.5 (±10.5) m constrained for Bristol in this work. As heat was released, the waste moved downward, an expected subsidence alteration for ETLFs, while high formation of leachate proceeded. Additionally, potential reactive wastes such as coal combustion residues are capable of starting strong exothermic reactions in a landfill [3]. A brief comparison of information available from quarry ETLFs is provided in Table S2 (Supporting Information) for landfilled depth, highest temperature, molar ratios of CO, H_2_, and CH_4_, the ratio of [CH_4_]/[CO_2_], excessive leachate production, settlement rate, and gas pressure. Indeed, the information from each case study is unique. For comparison, Table S2 also lists the information for conditions of a normal landfill.

Although the above studies summarized in Figure 3 through Figure 6 provided new insights about the landfill condition, the release of hazardous compounds to ground and surface waters was not investigated. A follow-up study of released toxicants by the landfill should be conducted to evaluate the environmental impact it has on water systems. The datasets of gaseous and leachate composition together with information on settlement patterns allowed the VDEQ to determine that the BVSW landfill evolved into an ETLF. Coal ash, officially dumped in the south end of the landfill (e.g., as registered in photographs from the years 2000 and 2004) [3] is a key precursor that could have catalyzed the ETLF formation. The landfill depth as of 2021 reached approximately 76–84 m [3]. Under the assumption of a constant filling rate per year (upward of 12 m y^−1^) based on the depth values from 1998 to 2021, coal ash deposited from 2000 to 2004 was added on top of waste landfilled at 8 to 26 m from the bottom [3]. These values correspond to depths from the top of the quarry (referenced to local ground level) of the coal ash disposed of ca. 79–100 m as of 15 June 2021 [3], where the source of elevated temperature should originate. As simulated by Hao et al. [63], the addition of even 10–20% of coal ash into the landfill can cause temperatures upward of 70–100 °C after 15 years. Excessive landfill heat accumulation occurs first by hydration to convert alkali metal oxides and alkaline earth metal oxides into hydroxides (e.g., reaction R5) and second by carbonation to transform the hydroxides into carbonates (e.g., reaction R6).


(R5) $$\text{CaO}(s)+{\text{H}}_{2}\text{O}(l)\to \text{Ca}(\text{OH}{)}_{2}(s)$$

(R6) $$\text{Ca}(\text{OH}{)}_{2}(s)+{\text{CO}}_{2}\to {\text{CaCO}}_{3}(s)+{\text{H}}_{2}\text{O}(l)$$

The pH of the landfill and its conditions play a large role in the progression of these reactions. In normal conditions, landfills will go through four phases: (1) aerobic, (2) anaerobic acid, (3) accelerated methane production, and (4) decelerated methane production [64]. As per reaction R2, aerobic processes in phase 1 will produce carbon dioxide and water while depleting oxygen. In phase 2, anaerobic acid reactions form carboxylic acids, decreasing the pH from 7.5 to ~6 (±0.3). Upon entering phase 3, carboxylic acids decrease in concentration while converted to methane via methanogens and the pH can rise to 7.9. Finally, in phase 4, a pH ≥ 8 can be reached as carboxylic acids become undetectable [64]. The previous sequence causes leachate within a normal landfill to have a pH between 7.5 and 8.5 [65].

However, when coal ash is introduced into a landfill as for the case of Bristol, normally in dry form [66], it eventually comes in contact with infiltrated water, causing hydration (reaction R5) and an increment in pH to 10–12 [67,68]. At pH 12, Ca(OH)_2_ is commonly found in equilibrium with gypsum (CaSO_4_•2H_2_O) [69]. Instead, the pH drops during the removal of hydroxides to produce carbonates (e.g., via reaction R6) [69]. As aged coal ash is present in the BVSW landfill, the leachate with high strength in CaCO_3_ should exhibit a pH of the waste of ~7.8–8.3 because a buffer system is formed with dissolved CO_2_ [68,69]. With the formation of an ETLF, a decrease in leachate pH to ~5.5–7.5 should be expected [70,71], e.g., as observed in Virginia and Ohio facilities [72]. The simultaneous decrease in alkalinity implies further formation of acidic organic compounds and carbonic acid from the high dissolution of CO_2_ common in such landfills [71].

The large amounts of heat released by hydration and carbonation reactions can enable waste combustion to proceed [33,34,63]. Hydration and carbonation reactions are typical for wastes containing calcium such as ash from municipal solid waste and coal combustion. The calcium oxide (CaO) content in municipal solid waste and coal ash can be as high as 50% and 40%, respectively [33]. For the hydration reaction of CaO, an enthalpy change of ${\Delta \text{H}=–1164\;\text{kJ kg}}^{-1}$ is produced, while the carbonation reaction has a ${\Delta \text{H}=–1719\;\text{kJ kg}}^{-1}$. Therefore, carbonation contributes nearly 1.5× the amount of heat as hydration reactions [63]. Carbonation reactions proceed at relevant depths (e.g., where coal ash was deposited for the BVSW landfill) with abundant anaerobic reactions due to the production of CO_2_. CO_2_ has been shown to reach stable concentrations between 40–60% at depths of 0.1–1 m [73,74] as the CH_4_/CO_2_ ratio reaches unity. Because the infiltration of water is common at the BVSW landfill, hydration reactions with coal ash are favored, with the entrapment of heat within the landfill. The water table, located at depths of ca. 38–46 m, inhibits the upward transfer of heat within the landfill while promoting its accumulation.

### Local Impact from Bristol’s ETLF Emissions.

Many of the compounds identified in the leachate and gas emissions of the Bristol ETLF can be toxic to residents in the surrounding community. Due to this public health concern, it has been recommended that the BVSW landfill is capped to reduce the continuous exposure to pollutants of the population [3]. The chronic effect of exposure to the most abundant hazardous pollutants recently identified in the BVSW landfill should be considered. For example, the detection of hydrogen sulfide (Figure 1) in the BVSW landfill contributes to explaining cases of respiratory irritation and the presence of unpleasant rotten egg odors, irritability, and headaches [75]. The presence of ammonia (Figure 2) and methyl mercaptan in the air can explain the perceived cleaning solutions and rotten cabbage smells, respectively, by nearby residents of the landfill. These compounds also create a burning sensation in the eyes, nose, throat, and respiratory tract [76,77].

Benzene, as a member of the BTEX family, shown in Figure 2, can negatively impact air quality in major cities, e.g., by contributing to the creation of smog [78]. Exposure to benzene, the most abundant chemical in the leachate and point source, results in eye and skin irritation [79]. Chronic exposure to benzene can cause depression of bone marrow followed by aplastic anemia. Further genetic damage induced by benzene causes leukemia and tumors [79,80]. In addition, many of the organic species detected in emissions from this landfill (e.g., ethanol, isopropanol, acetonitrile, acetone, and toluene) can contribute to eye and skin irritation, headaches, and nausea [81,82]. Moreover, the World Health Organization labeled chronic organic solvent intoxication syndrome as the daily exposure of people to such a combination of organic species with nervous system damage [83]. Thus, although the level of many VOCs detected in the BVSW landfill (e.g., alkanes, alkenes, alkynes, aromatics, hydrocarbons, and other oxygen-containing organic compounds) was apparently low, their cumulative concentration may have a public health impact [84], which is analyzed below in the health risk assessment section.

### Diel Cycling in the Bristol ETLF.

It must be first noted that gas emissions from landfills cannot be assumed to be constant over time due to diurnal variations in atmospheric pressure and winds, as seen often for compounds such as CH_4_ and CO_2_. A large evolution of CH_4_ and CO_2_ is produced from the degradation of organic matter represented in reaction R1 [85], which should occur in a normal landfill with a molar ratio of [CH_4_]/[CO_2_]~1 [11]. Other gases such as N_2_, O_2_, NH_3_, non-methane organic compounds (NMOCs), sulfides, H_2_, and CO are also typical of landfill emissions [12]. Specifically, NMOCs, like BTEX, hexane, and acrylonitrile, can result during the reactions between different waste materials [12]. Ultimately, fluxes of emitted volatiles over landfills can vary depending on precipitation, pressure, or wind patterns [85]. Depending on the composition of the waste (higher concentration of organics produces more gas), temperature (increase in temperature causes an increase in volatilization), and moisture content (increase in moisture increases bacterial decomposition) diurnal variations of volatile pollutants are expected in the BVSW landfill [12]. Therefore, the levels of H_2_S and NH_3_ from the BSVW landfill should vary in the corresponding time frame mainly due to moisture content and temperature within the landfill.

As is evident from the study monitoring VOCs described above [36], there are nighttime and daytime processes that play an important role in the emission, accumulation, and ventilation of toxic compounds in the air that people in the surrounding community to an ETLF breath. Among these processes, daytime and nighttime chemical reactions can be considered as well as changes in physical conditions of the planetary boundary layer (PBL) above the BVSW landfill. From the chemical viewpoint, daytime and nighttime reactions of the VOCs emitted by the Bristol ETLF vary with the respective formation of hydroxyl radical (HO) and two nocturnal nitrogen oxides, nitrate radical (NO_3_) and dinitrogen pentoxide (N_2_O_5_).

The combined nitrogen oxides (NO_x_ = NO + NO_2_) are widely available over the BVSW landfill atmosphere as they are emitted from power generation smokestacks and during the combustion of fossil fuels. The 1 h average molar ratio of NO_2_ in the Ohio Valley Region during the year 2021 was 39 (±13 ppb) [86]. Sunlight converts NO_2_ into nitric oxide (NO) and an oxygen atom (reaction R7), which can combine with O_2_ in the presence of a third body to form O_3_ (reaction R8):

(R7) $${\text{NO}}_{2}+hv\to \text{NO}+\text{O}$$

(R8) $$\text{O}+{\text{O}}_{2}\to {\text{O}}_{3}$$

Records for the U.S. Southeastern region where Bristol is located show 1 h average O_3_ levels of 60 (±4) ppb for the year 2021 [87]. Gas phase OH mainly originates from the reaction of a fraction of singlet oxygen atoms, resulting from the photolysis of ozone (O_3_), with water vapor to produce a daytime average average 2 × 10^5^ ≤ [OH] ≤ 1 × 10^6^ radicals cm^−3^:

(R9) $${\text{O}}_{3}+hv\to {\text{O}}_{2}+\text{O}$$

(R10) $$\text{O}+{\text{H}}_{2}\text{O}\to 2\;\text{OH}$$

Ozone can react readily with NO to yield NO_2_:

(R11) $$\text{NO}+{\text{O}}_{3}\to {\text{NO}}_{2}+{\text{O}}_{2}$$

In addition, nitrous oxide (N_2_O) emitted by the landfill is converted by O_3_ in NO contributing NO_x_ [88]. The emitted VOCs can also produce O_3_ in multistep reactions of smog formation under sunlight’s photons (hv) that involve the catalytic participation of OH,

(R12) $${\text{NO}}_{\text{x}}+\text{VOC}+h\nu \to {\text{O}}_{3}+\text{other products}$$

The other products in reaction R12 include the release of NO_2_ that is fed again to the pathway of O_3_ formation that creates OH. In more detail, the multistep reactions of emitted VOCs include OH oxidation by reaction R13 that proceeds through carbon center radicals (${\text{R}}^{•}$).

(R13) $$\text{VOC}+\text{OH}\to {\text{R}}^{•}+{\text{H}}_{2}\text{O}$$

The ${\text{R}}^{•}$ forms organic peroxides as depicted in reaction R14,

(R14) $${\text{R}}^{•}+{\text{O}}_{2}\to {\text{RO}}_{2}$$

which continue reacting with NO from the reactions above to form NO_2_ and a reactive alkoxy radical (RO) in reaction R15,

(R15) $${\text{RO}}_{2}+\text{NO}\to \text{RO}+{\text{NO}}_{2}$$

The fate of RO is to undergo isomerization and dissociation reactions resulting in ${\text{R}}^{•}$ or oxidation reactions that produce carbonyls that continue participating in smog formation. In addition, the available [OH(g)] can convert the emissions of H_2_S and NH_3_ from the Bristol ETLF in several steps into sulfur dioxide (SO_2_) and nitric oxide (NO), respectively. The daytime loss of such odoriferous species is logically explained, with the produced SO_2_ continuing its oxidation toward sulfate formation, while NO participates in reactions R11+R7 and R15. Thus, daytime reactions produce O_3_ and consume VOCs [88].

During nighttime, NO_2_ reacts with excess ozone to sequentially produce NO_3_ and N_2_O_5_ nitration agents,

(R16) $${\text{NO}}_{2}+{\text{O}}_{3}\to {\text{NO}}_{3}+{\text{O}}_{2}$$

(R17) $${\text{NO}}_{3}+{\text{NO}}_{2}⇌{\text{N}}_{2}{\text{O}}_{5}$$

The main pathway of N_2_O_5_ loss at night is its reaction with aqueous particles that remove NO_x_ from the gas phase,

(R18) $${\text{N}}_{2}{\text{O}}_{5}+{\text{H}}_{2}\text{O}(l)\to {\text{HNO}}_{3}(l)$$

and eventually the nitration agent by wet deposition. Nitrate radicals drive the dark transformation of VOCs to form oxidized VOCs (OVOCs) in reactions resulting in organic nitrates and peroxynitrates [89]:

(R19) $${\text{NO}}_{3}+\text{VOCs}\to \text{OVOCs}$$

Thus, nighttime reactions reduce O_3_ levels and produce OVOCs [90], while the destruction pathways of odoriferous reduced sulfur species are minimized.

### Entrainment and Ventilation of Air over the Bristol ETLF.

**Figure 8.**
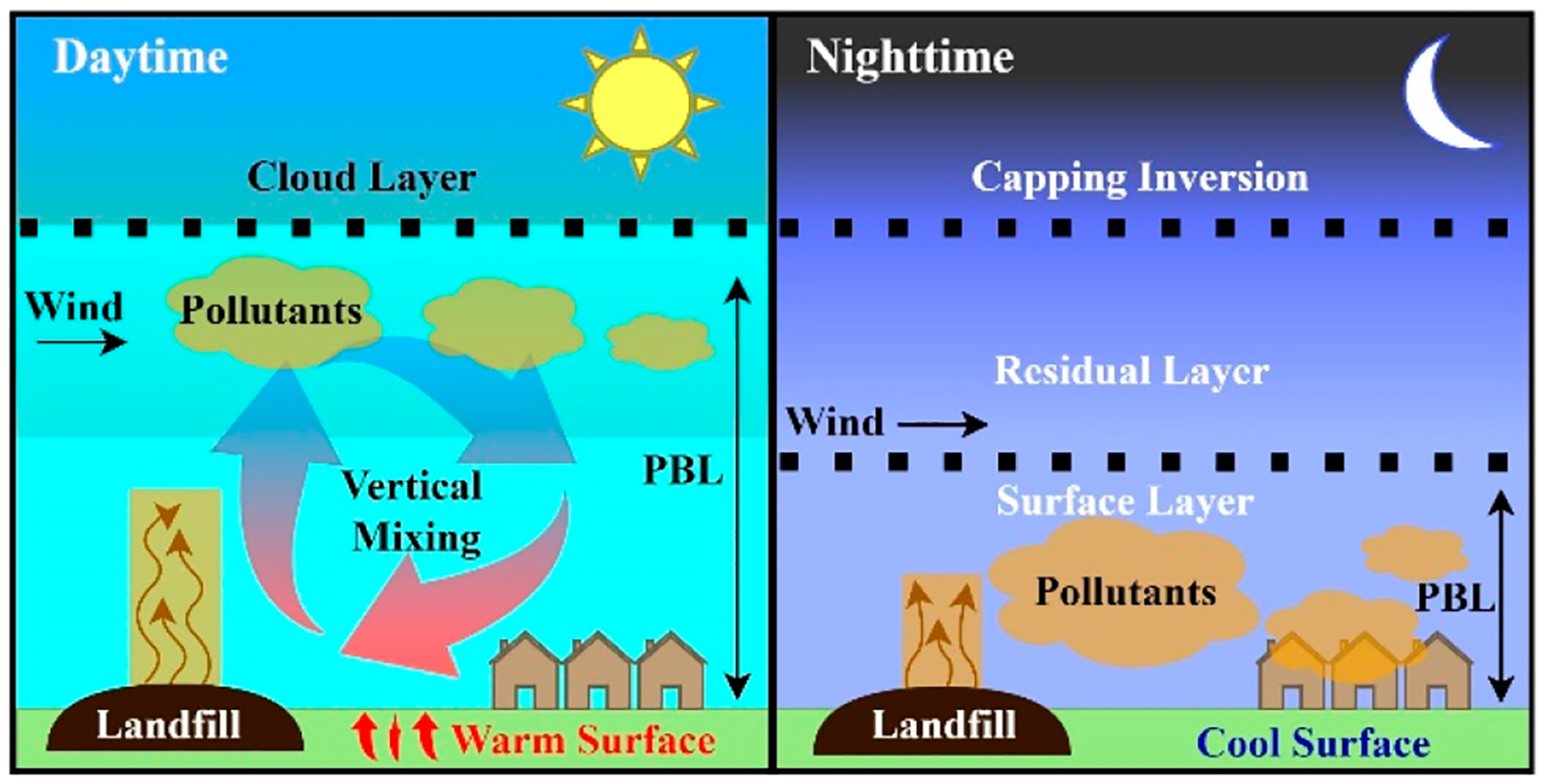
Diagram of (**Left**) daytime behavior of the Earth’s planetary boundary layer (PBL) allowing mixing of emitted air pollutants over the Bristol ETLF landfill and transport to the free troposphere. Under (**right**) nighttime conditions with a cooler surface, vertical mixing stops and the layer of air in contact with the surface becomes stable, allowing the accumulation of pollutants emitted to air at lower altitude.

The PBL layer over the BVSW landfill (Figure 8) is not static but instead changes dramatically over the course of 24 h. Considering midday as a reference for comparison, the atmospheric surface layer, directly in contact with the surface of the landfill, is highly oxidizing and alters the structure of pollutants. The PBL contains gases that are completely stirred in the compartment in less than 20 min, when solar radiation heats the surface and there is occasional upward air movement by convection of warm moist air. The resulting mixing with surrounding tropospheric air creates an entrainment zone as high as the cloud layer (e.g., ~1200 m altitude).

At sunset, the PBL starts to become stable as solar irradiance vanishes and the surface cools down, stopping convection and associated turbulent mixing of air in the surface layer that at night remains separate from the above stable residual layer. In consequence, the gaseous pollutants emitted from the Bristol ETLF are only mixed within this quasi-stagnant (altitude constrained) nocturnal PBL. Winds acting on the above residual layer create an unstable shear at the boundary that results in a small progressive expansion of the surface layer height with time at night. The height of this surface layer is typically ~10% of that in the original daytime mixed layer. Therefore, the pollutants are trapped near the surface at night, where their toxicological effect is more pronounced than during daytime.

As the sun rises and starts to reheat the surface, convection and mixing is developed again, with upward movement of air parcels until (e.g., from 8:30 to 10:30 am depending on the season among other conditions) the boundary layer is well mixed up to the cloud layer [88]. The overall effect of the resulting mixing is to allow the rapid ventilation and transport of the pollutants (that had been trapped at night) into the above free troposphere [88]. Wind patterns can influence the distance that pollutants are transported horizontally and their dilution in the free troposphere. Additionally, precipitation plays the most significant role in cleaning the pollutants from air by wet deposition. Rain events have been recognized by the Bristol community to wash the odors away [9]. The cloud formation process allows for an updraft of pollutants, which are eventually adsorbed and rained to the ground [88].

### Health Risk Assessment (*HRA*) Analysis.

The *HRA* of workers at the BVSW landfill is performed for the VOCs data in Figure 3 at the five locations studied [39]. Table 2 shows the weekly exposure (E) calculated with Equation (1) and the exposure ranking (ER) (Equation (2)) [39]. OEL values are presented in Table 3, where the range of ER values obtained are ranked as described in the methods section [39]. The results in Table 3 show that all VOCs, with the exception of benzene, belong to the EL1 category, presenting little risk, and that only exposure to benzene should be considered at the hot gas wells (EL2) and at the leachate tank and the point source (within EL5 category).

Table 4 reports the risk level (RL) of each VOC in Figure 3 obtained with Equation (3) [39], based on the hazard rate (HR) based on reference values [40–44]. In Table 4, compounds are described as catastrophically carcinogenic (HR=5), severely carcinogenic (HR=4), moderately carcinogenic (HR=3), minorly carcinogens (HR=2), and negligibly carcinogenic (HR=1) [91]. The calculated RL values can be categorized to have little (1.0–1.7), low (1.7–2.8), average (2.8–3.5), high (3.5–4.5), or very high (4.5–5) probability of occurrence [39]. Table 4 indicates that most of the VOCs detected occur with little probability of creating an exposure risk. However, the effects of benzene, ethylbenzene, ethanol, and isopropanol indicate a low risk level during a 4 h exposure. As concluded from the RL values, workers of the BVSW landfill experienced an increasing exposure to VOCs at the hot gas wells < the leachate tank < the point source.

The chronic daily intake of inhalation ($CDI_{\text{inhal}}$.) for each VOC obtained with Equation (4) [39,45] is listed in Table 5 and used to evaluate the health risk of exposed workers from the hazard quotient for inhalation of the VOCs ($HQ_{\text{inhal}}$.) obtained from Equation (5) [39,47]. Values of $HQ_{\text{inhal}}$. are listed in Table 6 and provide a non-cancerous risk assessment. Because all values of $HQ_{\text{inhal.}}<1$ in Table 6, it can be safely concluded that the workers of the BVSW landfill were not exposed to a non-cancer risk by the individual compounds. The bottom row of Table 6 provides the inhalation hazard index ($HI_{\text{inhal.}}$) obtained with Equation (6) [50]. While a potential risk from simultaneous exposure to all VOCs could be considerable if $HI_{\text{inhal.}}>1$, because the respective values in Table 6 are $HI_{\text{inhal.}}<1$, non-cancer health effects by the VOCs can be discarded.

The analysis above indicates the need to calculate the cancer risk (CR) for benzene only. Equation (7) is used to calculate $CR_{\text{Benzene}}$ during the 4 h exposure time, which accounts for the lowest exposure that can result in an increased carcinogenic risk to humans [39,47].

(7) $$CR_{\text{Benzene}}=CDI_{\text{inhal.}}\times CSF_{\text{Benzene}}$$

In Equation (7), $CSF_{\text{Benzene}}=0.055\;\text{kg day}\;{\text{mg}}^{-1}$ refers to the standardized cancer slope factor available [42]. The values obtained at the compost, hot gas wells, the existing landfill, the leachate tank, and the point source locations are 1.27 × 10^−7^, 1.26 × 10^−5^, 1.79 × 10^−7^, 2.44 × 10^−5^, and 7.41 × 10^−5^, respectively. Because $CR_{\text{Benzene}}<1\times 10^{-6}$ at the compost and existing landfill, there is no concern from exposure at these locations that a worker would develop cancer over a lifetime. However, at the hot gas wells, the leachate tank, and the point source, because $1\times 10^{-6}<CR_{\text{Benzene}}<1\times 10^{-4}$, further investigation by responsible agencies would be needed to investigate a potential risk for cancer development [50].

### Broader Implications of the BVSW ETLFs.

The information above serves as an example of the broad environmental implications not only to the Bristol Landfill but to other ETLFs. As discussed earlier, attention should be paid to the numerous factors that can facilitate an ETLF formation, such as deposited aluminum or coal ash, and the occurrence of surface fires [10]. Based on information from the United States Department of Energy, at least 1.1 million tons of aluminum waste is generated and placed in municipal landfills each year [11]. Moreover, about 40% of the 110 million tons of ash or combustion products generated annually is deposited into landfills [33].

Susceptible groups (including children, the elderly, and immunocompromised and asthmatic persons) [12] are more affected when exposed to hazardous gases and leachate emitted from ETLFs. One route for exposure to gases is possible after a breach in the landfill liner [3], allowing emissions to evolve and be dispersed through the surrounding community. A second pathway for exposure to gases is horizontal diffusion when the landfill is impermeable through compaction, the addition of daily soil covers, or the presence of highly saturated waste (usually with rainwater or leachate). Under such conditions, gases move horizontally and tend to accumulate in permeable subsurface areas such as utility corridors or basements in the neighborhood [12]. Thus, economically disadvantaged communities with poorly maintained infrastructure suffer more from the penetration of these gases through permeable surfaces in these underground areas. In addition, the accumulation of leachate from ETLFs is generally followed by runoff, which impacts surface waters and/or stormwater systems, resulting in an exposure path to pollutants. Once in the water system, the leachate can affect residential and commercial areas by altering drinking water quality.

Exposure to both gas and leachate pollution first and more intensively affects the residents in the surroundings of an ETLF, who are generally low-income communities compared to other groups. Tables S3 and S4 (Supporting Information) provide selected statistical data for urban areas in the United States where four ETLFs are located [96] and for their nearby elementary school, respectively [97–101]. For example, the population in Table S1 (Supporting Information) for Bristol, VA in 2022 was 16,975 of which 17.70% lived in poverty [102]. The race/ethnicity of Bristol’s (Virginia) population is listed as 88.4% White, 6.0% Black or African American, 5.3% two or more races, and 2.6% Hispanic or Latino [102]. Bristol (Virginia) also included 5.30%, 19.90%, and 22.10% of persons under 5 years, under 18 years, and over 65 years, respectively [102]. The race/ethnicity percentages for each city in Table S3 (Supporting Information) can be related to the same category for the closest elementary schools listed near the selected ETLFs in Table S4 (Supporting Information). For the example case of the BVSW landfill, Joseph Van Pelt Elementary and Highland View Elementary include 49.2% and 65.1% of economically disadvantaged persons (Table S4, Supporting Information), respectively [97,98]. Because children breathe more air, drink more water, and eat more food per pound of body weight than adults, this simple example is especially sensitive to a population subset that is more easily exposed to environmental contaminants [103]. In more detail, the respective race/ethnicity demographics for Joseph Van Pelt Elementary and Highland View Elementary are 76.7% and 72.0% White, 5.0% and 13.7% Black, 10.3% and 11.4% Multiple Races, 6.2% and 1.7% Hispanic, 1.7% and 0% Asian, and 0% and 1.1% American Indian [97,98]. Thus, the comparison of the broad city demographics (Table S3, Supporting Information) to those from the nearby schools (Table S3, Supporting Information) implies that the young population exposed to the ETLF pollution is from economically disadvantaged and diverse racial and ethnic origins.

The biggest barrier to understanding the extent of an ETLF development, such as that in Bristol, is data collection. The most common problem is the limited availability of long-term data regarding the leachate and the groundwater quality, air quality, and temperature of a landfill. The implementation of standard operation procedures by landfills to report the parameters for the leachate (e.g., BOD, chemical oxygen demand, pH, specific conductance, and total suspended solids) [70] and air quality should be implemented at large. Such information would facilitate the quick diagnostic of ETLFs and prevent other diverse and disadvantaged communities from experiencing similar problems in the future. Long-term data collection to understand the fate and transport of ETLF pollutants is also imperative for landfills to demonstrate that no harm is being caused to residents of neighboring communities when complex issues arise.

## 4. Conclusions

The results of the outdoor air monitoring study in the surrounding urban area to the BVSW landfill show that at nighttime, in the stagnant PBL, emissions of CH_3_SH, H_2_S, and NH_3_ have exceeded the threshold for odor detection [51–53], suggesting an explanation to cases of respiratory irritation, unpleasant odors, and headaches [75]. Instead, during the daytime, the PBL is well-mixed and ventilated. The same study registered three nighttime events with VOC levels reaching 13, 16, and 22 ppm [36]. The examination of VOCs emitted from five locations of the BVSW landfill allowed the calculation of the health risk index of exposed workers. The highest risk index associated with the point source and the leachate tank is indicative of the proximity of the source of elevated temperature and the direct transport of organic pollutants such as BTEX [3] through the copious leachate, respectively. The average molar ratio of CO (1978 (±226) ppm) and the average ratio [CH_4_]/[CO_2_] = 0.565 (±0.002) for the same five locations indicate that the BVSW landfill is affected by the elevated temperatures of 65 °C or larger in multiple sites, as supported also by the mapping analysis of gas well heads temperatures [3,14]. Elevated temperatures of 70 °C occur at gas well heads #64 and #65 in the northern portion and #66 and #67 in the southern portion, which also exhibit high oxygen content (>2%) [3]. Moreover, gas well heads #46 and #47 exhibit high temperatures that average 84 and 85 °C, respectively [38], independently of changing weather conditions. It was concluded that gas well heads #46, #47, #66, and #67 are clustered near the horizontal source of elevated temperature in the Southern portion, where coal ash had been deposited. Thus, the work establishes a connection between coal ash waste and its landfilled position to the initial transformation of the BVSW landfill into an ETLF. The key initiator for the BVSW ETLF development has been identified as municipal solid coal waste ash, which may be only common to the Waimanalo Gulch landfill in Hawaii [14,15], but different from most other ETLFs in Table S1 (Supporting Information) that included unknown industrial wastes, aluminum, steel slags, or combined precursors [3,10,14,16–19,21,22].

As a summary and guidance for local public health officers or members of an environmental protection agency/commission, ETLFs can be identified through the combined observation of elevated temperatures > 65 °C of gas head wells [14]; levels of methane <40% and carbon dioxide >50% that create molar ratios [CH_4_]/[CO_2_] ≪ 1 [10]; levels of carbon monoxide > 20 ppmv, hydrogen > 2% [10], and elevated ammonia relative to a normal landfill; a large production of volatile organic compounds [28,29]; and physical changes in the landfill such as rapid and severe waste settlement (>3% year^−1^), leachate seeps with a high BOD [10] and outbreaks [14], and an increase in gas pressure (>0.5 kPa) [10]. It is important to consider that an ETLF may not experience all the previous conditions simultaneously due to the progression of events a landfill undergoes during its transformation [70]. Moreover, a public health officer or environmental protection agency/commission member should also evaluate whether the key treatment technologies in the landfill have been undermined. (1) The integrity of a landfill liner can be visually inspected for any obvious signs of damage or wear, such as cracks, tears, or areas of subsidence [104]. In addition, inspecting the leak detection systems installed beneath the liner and performing a hydrologic evaluation of landfill performance (HELP) model can reveal liquid leaks that should not be there [105,106]. Additionally, the use of geophysical tests (e.g., electrical resistivity and ground penetrating radar) of the subsurface and regular chemical sampling and analysis of groundwater and leachate can help to detect any contaminant that should have not passed through the liner [104]. Finally, when applying increased pressure over the liner, a positive standardized test indicating a sudden pressure drop is also indicative of an integrity loss by the liner [107]. (2) The leachate collection and treatment systems should be checked frequently to detect unusual leachate flow rate changes and the presence of contaminants. (3) Similarly, it is important to inspect the gas extraction system (gas wells, piping, etc.) for signs of damage, flow changes, and gas composition variation (CH_4_, CO_2_, and O_2_ levels) with portable analyzers. The careful inspection of the landfill and its treatment technologies, as explained above, can indicate if the processes have been compromised and are not protecting the environment.

Initial suspicion of problems with a nearby landfill by surrounding communities may arise when multiple residents experience repeated events with strong chemical odors such as pungent decaying fish or rotten eggs, which may cause coughing and headaches. However, for a normal landfill, these symptoms usually subside once the exposure ends, but for an ETLF, they become pervasive. Based on the experience of Bristol’s residents [9], the first action step of affected persons is to document the odors, keeping a time and location record of the events and the weather conditions as details for supporting a report. Second, residents should report the odors to the authorities by contacting their local environmental protection agency/commission or health department, which can investigate the problem and take appropriate action. For example, each state in the United States [108], province in Canada [109], and country in the European Union [110] has an environmental protection agency that handles complaints related to air quality and odors, which can be accessed through the links in the references. State and country environmental agencies are a great point of contact to gain information on local agencies. However, federal/country agencies only directly handle problems with landfills and ETLFs (or other violations) that affect multiple cities, for extended violations beyond local jurisdictions, or for local cases that have remained unresolved and escalated to the federal level. Such organizations have direct online submission or downloadable forms to report environmental violations [111,112]. Importantly, some local health departments in the United States handle odor complaints and their contact information is available in the reference for the National Association of County and City Health Officials (NACCHO) directory [113].

As local environmental agencies/commissions know the laws and local regulations, they are often the most useful organizations to advise about specific environmental problems. Directories on state government websites [114] often list the departments that handle environmental and health concerns. When reporting an environmental violation to these agencies, the following information will be required upon submission: name of the facility, address, time of the violation, location of the violation, systems that violations have affected (air, water, land, etc.), and a description of the event. Personal information may be requested by these agencies if follow-up is needed. Furthermore, the use of major search engines (e.g., Googe, Bing, etc.) can help direct residents to their local government websites for more localized issues and gain access to the contact information of departments that handle public complaints. Similarly, information about nearby landfills and ETLFs can be gained using such search engines. An additional (but not substitute) measure is for residents to directly reach out to the landfill operator, which may lead to a quicker resolution as they might be unaware of the issue and can take steps to mitigate the odor. Residents can form or engage with community groups to collectively address the issue. Community advocacy can be powerful in prompting action from local authorities and landfill operators and contribute to keeping residents informed. If the odors and problems persist in a way that significantly impacts the quality of life of residents, the final step consists of consulting a lawyer to take legal action.

Potential strategies for the Bristol ETLF should be considered in the context of the data presented. The state of Virginia claimed a lack of compliance by the BVSW landfill with the air pollution control permit and solid waste management. The BVSW landfill received several notices of violation through 2020–2022, including (1) landfill operating temperatures exceeded 65 °C for 15 days or more [115–117]; (2) oxygen levels exceeded 5% for 15 days or more [115,117]; (3) positive wellhead pressures for 15 days or more [115,117]; (4) methane concentrations exceeded 500 ppm [116]; (5) air pollution control laws and regulations [115]; (6) gradient control pumps for leachate were inoperable causing the escape of leachate into stormwater systems [116,117]; (7) soil coverage was not maintained for the active working face of the landfill [116]; and (8) monthly inspections, emission records, and other documentation were not monitored or recorded [115,116].

Following the confirmed diagnosis of the BVSW landfill transformation into an ETLF, mitigation strategies must be implemented to protect the public health and well-being of the community. The three options that should be considered are (1) mitigating odors, (2) limiting waste disposal, and (3) closing and capping the landfill permanently [3]. The BVSW landfill has been closed and is not accepting waste since September 2022, which requires transporting community waste to another landfill in Tennessee [5]. In addition, the installation of an air monitoring network was ordered to monitor H_2_S, other sulfides, and VOCs [118]. Other remedial work such as waste temperature monitoring, gas collection, sidewall odor mitigation, upgrading wells and pumps, leachate extraction and monitoring, intermediate cover, stormwater management, and intensive mapping of the landfill are required from the attorney general’s consent decree [119].

Finally, an important learning from this case study is that the high temperatures (T > 80 °C) experienced by the BVSW landfill could have seriously compromised its environmental control systems. In consequence, on multiple occasions, the BVSW landfill has released excessive amounts of leachate runoff (typical of ETLFs) into groundwater and surface water systems due to malfunctioning pumps. The released leachate could be considered as hazardous, affecting not only the water systems but also the residents of Bristol [9]. However, current data do not conclusively indicate that toxicological levels were exceeded under the current no observed adverse effect level (NOAEL) and lowest observed adverse effect level (LOAEL) from EPA regulations.

## Supplementary Material

Supplementary Material

The following supporting information (PDF) is available free of charge on the website https://www.mdpi.com/article/10.3390/environments11090201/s1: Table S1 with a list of ETLFs, Table S2 with a comparison of ETLFs, Table S3 with the demographics of selected cities with ETLFs, and Table S4 with the demographics of elementary schools near ETLFs in Table S3.

## Figures and Tables

**Table 1.** BVSW landfill gas well head temperature increase and oxygen molar ratio [3].

Southern Landfill		
Gas Well Number	Oxygen Molar Ratio (%)	Temperature Increase (°C) (Min–Max)
**35**	0.0–3.6	18 (33–51)
**39**	9.7–21.2	18 (20–38)
**40**	2.0–18	35 (4–39)
**46**	0.5–18	47 (21–68)
**47**	0.0–1.1	25 (27–52)
**66**	1.2–7.3	36 (34–70)
**67**	1.2–6.7	16 (54–70)
**68**	2.6–3.9	15 (34–49)
Northern Landfill		
**29**	1.0–20.5	24 (15–39)
**29R**	0.5–20.5	22 (27–49)
**31R**	0.1–2.7	30 (39–69)
**32**	0.1–2.6	12 (18–30)
**32R**	0.4–2.7	11 (41–52)
**33**	0.5–12.0	44 (10–54)
**37**	0.0–0.9	29 (27–56)
**62**	0.0–10.0	33 (21–54)
**64**	1.2–9.2	13 (57–70)
**65**	1.2–7.3	36 (34–70)

**Table 2.** Concentration of individual VOCs in the air $\left(C_{\text{air}}\right)$ in locations of the BVSW landfill [37] and calculated weekly exposure (E).

Compound	$C_{\text{air}}\;\left(\mu \text{g}\;{\text{m}}^{-3}\right)$					$E\left(\text{mg}\;{\text{m}}^{-3}\right)$				
	Compost	Hot Gas Wells	Existing Landfill	Leachate Tank	Point Source	Compost	Hot Gas Wells	Existing Landfill	Leachate Tank	Point Source
**Benzene**	0.802	79.4	1.13	154	467	0.0011	0.1112	0.0016	0.2156	0.6538
**Toluene**	1.43	18.1	2.07	28	36.7	0.0020	0.0253	0.0029	0.0392	0.0514
**Ethylbenzene**	ND	13.2	0.832	25.3	24.7	0.0000	0.0185	0.0012	0.0354	0.0346
**m/p-Xylene**	1.65	12.8	3.03	21.4	36.2	0.0023	0.0179	0.0042	0.0300	0.0507
**o-Xylene**	0.56	4.33	0.934	7.26	15.9	0.0008	0.0061	0.0013	0.0102	0.0203
**Ethanol**	6.28	69.2	8.5	85.5	46.8	0.0088	0.0969	0.0119	0.1197	0.0655
**Isopropanol**	2.92	19.8	3.58	28.2	5.24	0.0041	0.0277	0.0050	0.0395	0.0073
**Acetone**	5.67	45.6	7.03	60.6	17.3	0.0079	0.0638	0.0098	0..0848	0.0242
**2-Butanone**	0.546	21	0.77	32.4	4.3	0.0008	0.0294	0.0011	0.0454	0.0060
**Propylene**	3.68	14.4	7.45	22.1	45.4	0.0052	0.0202	0.0104	0.0309	0.0636
**Heptane**	0.799	2.34	0.65	3.05	3.66	0.0011	0.0033	0.0009	0.0043	0.0051
**Hexane**	49.1	10.4	1.47	36.1	36.2	0.0687	0.0146	0.0021	0.0505	0.0507
**Acetonitrile**	0.611	5.06	27.4	0.295	4.23	0.0009	0.0071	0.0384	0.0004	0.0059
**Ethyl Acetate**	1.43	4.2	1.93	5.13	2.61	0.0020	0.0059	0.0027	0.0072	0.0037
**Freon 11**	1.33	1.66	1.39	1.68	1.91	0.0019	0.0023	0.0019	0.0024	0.0027
**Freon 12**	2.68	2.89	2.72	2.69	2.95	0.0038	0.0040	0.0038	0.0038	0.0041
**Chloromethane**	1.24	2.11	1.27	2.09	1.88	0.0017	0.0030	0.0018	0.0029	0.0026

ND: Not detected.

**Table 3.** Occupational exposure limit (OEL) [40], exposure ranking (ER) of individual VOCs in locations of the BVSW landfill and assigned exposure level (EL).

Compound	$\text{OEL}(\text{mg}\;{\text{m}}^{-3})$	Compost	Hot Gas Wells	ER Existing Landfill	Leachate Tank	Point Source	Compost	Hot Gas Wells	EL Existing Landfill	Leachate Tank	Point Source
**Benzene**	0.1	0.0112	1.1116	0.0158	2.1560	6.5380	1	2	1	5	5
**Toluene**	100	0.0000	0.0003	0.0000	0.0004	0.0005	1	1	1	1	1
**Ethylbenzene**	100	0.0000	0.0002	0.0000	0.0004	0.0003	–	1	1	1	1
**m/p-Xylenes**	100	0.0000	0.0002	0.0000	0.0003	0.0005	1	1	1	1	1
**o-Xylenes**	100	0.0000	0.0001	0.0000	0.0001	0.0002	1	1	1	1	1
**Ethanol**	1000	0.0000	0.0001	0.0000	0.0001	0.0001	1	1	1	1	1
**Isopropanol**	400	0.0000	0.0001	0.0000	0.0001	0.0000	1	1	1	1	1
**Acetone**	250	0.0000	0.0003	0.0000	0.0003	0.0001	1	1	1	1	1
**2-Butanone**	200	0.0000	0.0001	0.0000	0.0002	0.0000	1	1	1	1	1
**Propylene**	100	0.0001	0.0002	0.0001	0.0003	0.0006	1	1	1	1	1
**Heptane**	85	0.0000	0.0000	0.0000	0.0001	0.0001	1	1	1	1	1
**Hexane**	50	0.0014	0.0003	0.0000	0.0010	0.0010	1	1	1	1	1
**Acetonitrile**	20	0.0000	0.0004	0.0019	0.0000	0.0003	1	1	1	1	1
**Ethyl Acetate**	400	0.0000	0.0000	0.0000	0.0000	0.0000	1	1	1	1	1
**Freon 11**	1000	0.0000	0.0000	0.0000	0.0000	0.0000	1	1	1	1	1
**Freon 12**	1000	0.0000	0.0000	0.0000	0.0000	0.0000	1	1	1	1	1
**Chloromethane**	100	0.0000	0.0000	0.0000	0.0000	0.0000	1	1	1	1	1

**Table 4.** Identified IRAC group, reference (Ref.) for group assignment to hazard rate (HR) and risk level (RL) from individual VOCs during 4 h exposure in locations of the BVSW landfill.

Compound	*IRAC Group*	Ref.	HR	RL				
				Compost	Hot Gas Wells	Existing Landfill	Leachate Tank	Point Source
**Benzene**	Group 1	[40,41]	5	2.24	3.16	2.24	5.00	5.00
**Toluene**	Group 3	[40,41]	2	1.41	1.41	1.41	1.41	1.41
**Ethylbenzene**	Group 2B	[40,41]	3	–	1.73	1.73	1.73	1.73
**m/p-Xylenes**	Group 3B	[40,41]	1	1.00	1.00	1.00	1.00	1.00
**o-Xylenes**	Group 3B	[40,41]	1	1.00	1.00	1.00	1.00	1.00
**Ethanol**	Group 1	[40,41]	5	2.24	2.24	2.24	2.24	2.24
**Isopropanol**	Group 1	[40,41]	5	2.24	2.24	2.24	2.24	2.24
**Acetone**	A4	[40,43]	2	1.41	1.41	1.41	1.41	1.41
**2-Butanone**	Group D	[40,42]	1	1.00	1.00	1.00	1.00	1.00
**Propylene**	Group 3B	[40,41]	1	1.00	1.00	1.00	1.00	1.00
**Heptane**	Group D	[40,42]	1	1.00	1.00	1.00	1.00	1.00
**Hexane**	Group D	[40,42]	1	1.00	1.00	1.00	1.00	1.00
**Acetonitrile**	A4	[40,43]	2	1.41	1.41	1.41	1.41	1.41
**Ethyl Acetate**	Group 3B	[40,44]	1	1.00	1.00	1.00	1.00	1.00
**Freon 11**	A4	[40,43]	2	1.41	1.41	1.41	1.41	1.41
**Freon 12**	A4	[40,43]	2	1.41	1.41	1.41	1.41	1.41
**Chloromethane**	Group D	[40,42]	1	1.00	1.00	1.00	1.00	1.00

**Table 5.** Chronic daily intake by inhalation $\left(CDI_{\text{inhal.}}\right)$ based on concentrations in air $\left(C_{\text{air}}\right)$ in Table 2 at the locations of the BVSW landfill.

Compound	${\text{CDI}}_{\text{inhal.}}({\text{mg kg}}^{-1}\;{\text{day}}^{-1})$				
	Compost	Hot Gas Wells	Existing Landfill	Leachate Tank	Point Source
**Benzene**	2.31 × 10^−6^	2.29 × 10^−4^	3.26 × 10^−6^	4.44 × 10^−4^	1.35 × 10^−3^
**Toluene**	4.12 × 10^−6^	5.22 × 10^−5^	5.97 × 10^−6^	8.07 × 10^−5^	1.06 × 10^−4^
**Ethylbenzene**	–	3.81 × 10^−5^	2.40 × 10^−6^	7.29 × 10^−5^	7.12 × 10^−5^
**m/p-Xylenes**	4.76 × 10^−6^	3.69 × 10^−5^	8.74 × 10^−6^	6.17 × 10^−5^	1.04 × 10^−4^
**o-Xylene**	1.61 × 10^−6^	1.25 × 10^−5^	2.69 × 10^−6^	2.09 × 10^−5^	4.58 × 10^−5^
**Ethanol**	1.81 × 10^−5^	2.00 × 10^−4^	2.45 × 10^−5^	2.47 × 10^−4^	1.35 × 10^−4^
**Isopropanol**	8.42 × 10^−6^	5.71 × 10^−5^	1.03 × 10^−5^	8.13 × 10^−5^	1.51 × 10^−5^
**Acetone**	1.63 × 10^−5^	1.31 × 10^−4^	2.03 × 10^−5^	1.75 × 10^−4^	4.99 × 10^−5^
**2-Butanone**	1.57 × 10^−6^	6.05 × 10^−5^	2.22 × 10^−6^	9.34 × 10^−5^	1.24 × 10^−5^
**Propylene**	1.06 × 10^−5^	4.15 × 10^−5^	2.15 × 10^−5^	6.37 × 10^−5^	1.31 × 10^−4^
**Heptane**	2.30 × 10^−6^	6.75 × 10^−6^	1.87 × 10^−6^	8.79 × 10^−6^	1.06 × 10^−5^
**Hexane**	1.42 × 10^−4^	3.00 × 10^−5^	4.24 × 10^−6^	1.04 × 10^−4^	1.04 × 10^−4^
**Acetonitrile**	1.76 × 10^−6^	1.46 × 10^−5^	7.90 × 10^−5^	8.51 × 10^−7^	1.22 × 10^−5^
**Ethyl Acetate**	4.12 × 10^−6^	1.21 × 10^−5^	5.56 × 10^−6^	1.48 × 10^−5^	7.53 × 10^−6^
**Freon 11**	3.83 × 10^−6^	4.79 × 10^−6^	4.01 × 10^−6^	4.84 × 10^−6^	5.51 × 10^−6^
**Freon 12**	7.73 × 10^−6^	8.33 × 10^−6^	7.84 × 10^−6^	7.76 × 10^−6^	8.51 × 10^−6^
**Chloromethane**	3.58 × 10^−6^	6.08 × 10^−6^	3.66 × 10^−6^	6.03 × 10^−6^	5.42 × 10^−6^

**Table 6.** Hazard quotient for inhalation $\left(HQ_{\text{inhal.}}\right)$ in the BVSW landfill locations, daily reference dose (RfD), and hazard index $\left(HI_{\text{inhal.}}\right)$.

Compound	$RfD({\text{mg kg}}^{-1}\;{\text{day}}^{-1})$	$HQ_{\text{inhal.}}$ during 4 h				
		Compost	Hot Gas Wells	Existing Landfill	Leachate Tank	Point Source
**Benzene**	0.0040 ^a^	5.78 × 10^−4^	5.72 × 10^−2^	8.15 × 10^−4^	1.11 × 10^−1^	3.37 × 10^−1^
**Toluene**	0.0800 ^a^	5.15 × 10^−5^	6.52 × 10^−4^	7.46 × 10^−5^	1.01 × 10^−3^	1.32 × 10^−3^
**Ethylbenzene**	0.1000 ^a^	–	3.81 × 10^−4^	2.40 × 10^−5^	7.29 × 10^−4^	7.12 × 10^−4^
**m/p-Xylenes**	0.2000 ^a^	2.38 × 10^−5^	1.85 × 10^−4^	4.37 × 10^−5^	3.09 × 10^−4^	5.22 × 10^−4^
**o-Xylene**	0.2000 ^a^	8.07 × 10^−6^	6.24 × 10^−5^	1.35 × 10^−5^	1.05 × 10^−4^	2.29 × 10^−4^
**Ethanol**	38.45 ^b,f^	4.71 × 10^−7^	5.19 × 10^−6^	6.37 × 10^−7^	6.41 × 10^−6^	3.51 × 10^−6^
**Isopropanol**	0.0243 ^c,f^	3.47 × 10^−4^	2.35 × 10^−3^	4.25 × 10^−4^	3.35 × 10^−3^	6.22 × 10^−4^
**Acetone**	0.9000 ^a^	1.82 × 10^−5^	1.46 × 10^−4^	2.25 × 10^−5^	1.94 × 10^−4^	5.54 × 10^−5^
**2-Butanone**	0.6000 ^a^	2.62 × 10^−6^	1.01 × 10^−4^	3.70 × 10^−6^	1.56 × 10^−4^	2.07 × 10^−5^
**Propylene**	0.3643 ^d,f^	2.91 × 10^−5^	1.14 × 10^−4^	5.90 × 10^−5^	1.75 × 10^−4^	3.59 × 10^−4^
**Heptane**	0.4857 ^a,f^	4.74 × 10^−6^	1.39 × 10^−5^	3.86 × 10^−6^	1.81 × 10^−5^	2.17 × 10^−5^
**Hexane**	0.3000 ^e^	4.72 × 10^−4^	1.00 × 10^−4^	1.41 × 10^−5^	3.47 × 10^−4^	3.48 × 10^−4^
**Acetonitrile**	0.0073 ^a,f^	2.42 × 10^−4^	2.00 × 10^−3^	1.08 × 10^−2^	1.17 × 10^−4^	1.67 × 10^−3^
**Ethyl Acetate**	0.9000 ^a^	4.58 × 10^−6^	1.35 × 10^−5^	6.18 × 10^−6^	1.64 × 10^−5^	8.36 × 10^−6^
**Freon 11**	0.3000 ^a^	1.28 × 10^−5^	1.60 × 10^−5^	1.34 × 10^−5^	1.61 × 10^−5^	1.84 × 10^−5^
**Freon 12**	0.2000 ^a^	3.86 × 10^−5^	4.17 × 10^−5^	3.92 × 10^−5^	3.88 × 10^−5^	4.25 × 10^−5^
**Chloromethane**	0.0011 ^a,f^	3.27 × 10^−3^	5.57 × 10^−3^	3.35 × 10^−3^	5.51 × 10^−5^	4.96 × 10^−3^
${\text{HI}}_{\text{inhal.}}$		0.01	0.07	0.02	0.12	0.35

Source:

a ref. [42],

b ref. [92],

c ref. [93],

d ref. [48],

e ref. [94], and

f calculated value from reference concentrations as recommended in ref. [95].

## Data Availability

All source data are publicly available through the links provided in the references. All contributions presented in the study are included in the article/Supplementary Material; further inquiries can be directed to the corresponding author.

## Abbreviations

BOD: biological oxygen demand
BTEX: benzene, toluene, ethylbenzene, and xylenes
BVSW: Bristol Virginia solid waste
EPA: Environmental Protection Agency
ETLF: elevated temperature landfill
*hν*: sunlight’s photons
OVOCs: oxidized volatile organic compounds
PBL: planetary boundary layer
ppm: parts per million by volume
T: temperature
VDEQ: Virginia Department of Environmental Quality
VOCs: volatile organic compounds
ΔH: enthalpy change
