# Supplementary material for: Coal Ash Triggers an Elevated Temperature Landfill Development: Lessons from the Bristol Virginia Solid Waste Landfill Neighboring Community
Source: Environments (Basel). Author manuscript; Available in PMC 2026 Apr 1. (PMC13038288; doi:10.3390/environments11090201)
Supplement: Supplementary Material [file NIHMS2150753-supplement-Supplementary_Material.pdf]

Supporting Information For:

Coal Ash Triggers an Elevated Temperature Landfill Development: Lessons from the Bristol Virginia Solid Waste Landfill Neighboring Community

Environments

Year 2024

*Reagan Patton Witt and Marcelo I. Guzman**

Department of Chemistry, University of Kentucky, Lexington, Kentucky 40506, United States

Corresponding author's email: marcelo.guzman@uky.edy

Table S1. List of Elevated Temperature Landfills (ETLFs)

Case #	**Name**	**Location**	**Country**	**Precursor**	**Ref.**
**1**	Bristol Landfill [ε,φ]	Bristol, Tennessee	United States	Municipal solid waste ash	1
**2**	Bridgeton Landfill [ε,φ]	Bridgeton, Missouri	United States	Unknown industrial waste	2,3
**3**	Hillside Landfill [ε, φ]	Hillside, Illinois	United States	Coal ash, lime, aluminum	4
**4**	Countrywide Landfill [ε, φ]	East Sparta, Ohio	United States	Aluminum	1,2
**5**	Waimanalo Gulch Landfill [φ, φ]	Oahu, Hawaii	United States	Municipal solid waste ash	2,5
**6**	Noble Road Landfill	Shiloh, Ohio	United States	Steel slag	2,6
**7**	Middle Point Landfill [ε, φ]	Middle Point, Tennessee	United States	Aluminum	2,7
**8**	Rumpke Landfill [ε]	Cincinnati, Ohio	United States	Unknown industrial waste	2,8
**9**	Twenty landfills with elevated temperature[, φ]	Florida	United States	Municipal solid waste ash (7 out of the 20 sites)	9
**10**	Brantley Landfill	Island, KY	United States	Salt cake fines	10
**11**	Alumitech of Wabash[, φ]	Wabash, Indiana	United States	Aluminum	11
**12**	RAMCO waste disposal site[, φ]	Dallesport, Washington	United States	Aluminum	7
**13**	Lon Landfill	Negev	Israel	Construction	12
**14**	Alonim Landfill	Coastal Plain	Israel	Construction	12
**15**	Kfar Gidon Landfill	Galil	Israel	Industrial	12
**16**	Abu Dis Landfill	Judea Hills	Israel	Construction	12
**17**	Sunnhordland Intermunicipal	Sunnhordaland	Norway	Industrial and construction	13
**18**	Tagarades	Thessaloniki	Greece	Unknown	14
**19**	Tokyo Port Landfill	Tokyo	Japan	Water infiltration	15
**20**	Jeleeb Al-Shuyoukh Landfill	Jeleeb Al-Shuyoukh	Kuwait	Unknown	16

[ε] Quarry site. [φ] The presence of coal ash, aluminum dross, or incineration waste has been indicated to contribute to the creation of an ETLF.

Table S2. Comparison of Selected Quarry ETLFs from Table S1.

Landfill	Bristol[1,17,18]	Bridgeton[1,3]	Hillside[1,3,19]	Normal Conditions[3,20,21]
**Landfilled Depth (m)**	84	98	78	Varies per permit
**Highest Temperature (°C)**	85	135	95	< 65 ºC
**Maximum CO Molar Ratio (ppm)**	2,250*	> 1,500	2,500	< 20
**Maximum H_2 Molar Ratio (%)**	0.4*	NDA	20	< 1
**Maximum CH_4 Molar Ratio (%)**	0.179*	~1	NDA	60
**Maximum Ratio $[CH_4]:[CO_2]$**	0.567*	NDA	0.3	~1
**Excessive Leachate Production**	Yes	Yes	Yes	No
**Maximum Settlement Rate (% $year^{-1}$)**	> 3	NDA	5.7	3
**Gas Pressure (kPa)**	> 5	NDA	14	< 5

NDA: No data available. *Contribution of air is suspected during sampling

Table S3. Population Demographics in 2022 of Selected Cities with ETLFs[22]

	Bristol, Virginia	Bristol, Tennessee	Honolulu, Hawaii	Hillside, Illinois	Bridgeton, Missouri	United States
**Population**	16,975	27,705	995,638	8,005	11,338	333,287,557
**Females**	52.6%	53.2%	49.4%	49.2%	53.7%	50.4%
**Children under 5 years old**	5.3%	5.7%	5.6%	6.5%	5.3%	5.6%
**Children under 18 years old**	19.9%	21.6%	20.5%	18.4%	21.9%	21.7%
**Persons over 65 years old**	22.1%	17.9%	19.6%	17.2%	21.2%	17.3%
**Live in poverty**	17.7%	19.6%	10.0%	6.0%	9.3%	11.5%
**White**	88.4%	91.3%	21.2%	28.6%	68.6%	75.5%
**Black or African American**	6.0%	2.8%	2.8%	44.9%	20.7%	13.6%
**Two or More Races**	5.3%	4.6%	23.2%	2.8%	3.5%	3.0%
**American Indian**	0%	0.1%	0.3%	0.7%	0.1%	1.3%
**Asian**	0.3%	0.5%	42.6%	2.5%	3.3%	6.3%
**Pacific Islander**	0%	0%	9.9%	0%	0%	0.3%
**Hispanic or Latino**	2.6%	2.9%	10.3%	33.4%	9.2%	19.1%

Table S4. Population Demographics for Elementary Schools near Case Studies

	Joseph Van Pelt[23]	Highland View[24]	Barbers Point[25]	Hillside[26]	Garret
**Nearby Landfill Address**	2125 Shakesville Rd, Bristol, VA 24201		92-460 Farrington Hwy, Kapolei, HI 96707	4100 N Frontage Rd, Hillside, IL 60162	13570 St Charles Rock Rd, Bridgeton, MO 63044
**White**	76.1%	72.0%	13.6%	2.8%	29.7%
**Black**	5.0%	13.7%	3.7%	41.1%	54.6%
**Multi Racial**	10.3%	11.4%	17.1%	2.3%	3.8%
**Hispanic**	6.2%	1.7%	24.3%	50.5%	9.2%
**Asian**	1.7%	0%	10.1%	1.9%	1.7%
**American Indian**	0%	1.1%	0.8%	1.4%	0.7%
**Hawaiian Pacific Islander**	0%	0%	30.3%	0%	0.3%
**Economically Disadvantaged[a]**	49.2%	65.1%	71%	61.8%	52%

[a] A student is reported as economically disadvantaged if he or she meets any one of the following criteria: Is eligible for free/reduced meals; receives temporary assistance for needy families; is eligible for Medicaid; or is a migrant or is experiencing homelessness.

References

1. Benson, C.; Chiado, E.; Gardner, R.; Novak, J.; Sperling, T.; Stark, T.; Thalhamer, T.; Widdowson, M.; Williams, M.; Wyatt, E. *Expert Panel Report: Bristol Integrated Solid Waste Management Facility, Bristol, Virginia*; Virginia Polytechnic Institute: 2022, (accessed on 1 July 2023).
2. Environmental Protection Agency. When Does a Municipal Solid Waste Landfill Become an Elevated Temperature Landfill (ETLF)? Available online: https://www.epa.gov/system/files/documents/2022-04/elevated-temperature-landfills-factsheet-1.pdf (accessed on
3. Jafari, N.H.; Stark, T.D.; Thalhamer, T. Spatial and temporal characteristics of elevated temperatures in municipal solid waste landfills. *Waste Managem.* **2017**, *59*, 286-301.
4. Illinois First Judicial District. The Village of Hillside, Illinois v. Congress Development Company. Available online: https://www.illinoiscourts.gov/Resources/0ceed425-8d71-47b5-a5dd-f07032ec20b9/1131253_R23.pdf (accessed on 9 November 2023).
5. Higuchi, D. EPA reaches agreement over Waimanalo Gulch Landfill fire threat / $1.1 million penalty for Clean Air Act violations. Available online: https://www.epa.gov/archive/epapages/newsroom_archive/newsreleases/78dd2c0dbd481e7685257b20006f9325.html (accessed on
6. Countywide Recycling and Disposal Facility. Final Report: Work Activities Pursuant to Administrative Agreement and Order on Consent for Removal Action (AOC). Available online: https://response.epa.gov/sites/3944/files/aoc%20final%20report%20complete%2010-22-09.pdf (accessed on 9 November 2023).
7. Washington State Department of Ecology. Interim Remedial Action Plan RAMCO Aluminum Waste Disposal Site Port of Klicktat Industrial Park Dallesport, Washington. Available online: https://apps.ecology.wa.gov/gsp/DocViewer.ashx?did=889 (accessed on 9 November 2023).
8. Ohio Environmental Protection Agency. Rumpke Landfill Update Hamilton County. Available online: https://response.epa.gov/sites/5373/files/Rumpke%20Landfill%20Fact%20Sheet%20May%202010.pdf (accessed on 9 November 2023).

9. Reinhart, D.; Joslyn, R.; Emrich, C.T. Characterization of Florida, US landfills with elevated temperatures. *Waste Managem.* **2020**, *118*, 55-61.

10. Environmental Protection Agency. EPA Superfund Record of Decision: Brantley Landfill Site, Island, KY. Available online: https://nepis.epa.gov/Exe/ZyPURL.cgi?Dockey=9100L6ZD.TXT (accessed on 9 November 2023).

11. Indiana Department of Environmental Management. Cause No. A-4642 and Cause No. SW-416 Alumitech of Wabash, Inc. Available online: https://www.in.gov/idem/oe/cause/AO/8776-S.htm (accessed on 9 November 2023).

12. Frid, V.; Doudkinski, D.; Liskevich, G.; Shafran, E.; Averbakh, A.; Korostishevsky, N.; Prihodko, L. Geophysical-geochemical investigation of fire-prone landfills. *Environ. Earth Sci.* **2010**, *60*, 787-798.

13. Øygard, J.K.; Måge, A.; Gjengedal, E.; Svane, T. Effect of an uncontrolled fire and the subsequent fire fight on the chemical composition of landfill leachate. *Waste Managem.* **2005**, *25*, 712-718.

14. Nikolaou, A.D. Environmental management and landfill fire accidents. *J. Environ. Protec. Ecol.* **2008**, *9*, 830-834.

15. Hoor, A.; Rowe, R.K.; Pollard, A. A method for reducing the temperature of landfill liners in MSW landfills. In Proceedings of the Proc. Global Waste Management Symp. (CD-ROM), 2008.

16. Gill, J.; Faisal, K.; Shaker, A.; Yeung Yan, W. Detection of Waste dumping location in landfill using multi-temporal Landsat thermal images. *Waste Manage Res* **2019**, *37*, 386-393.

17. TetraTech. Final Air Monitoring Report. Available online: https://response.epa.gov/sites/15220/files/DTN0272_Final%20Air%20Monitoring%20Report_Bristol%20Air_Rev0-%20SIGNED%20MPB_REDACTED.pdf (accessed on 4 March 2022).

18. Trinity Consultants. Air Sampling at Bristol, Virginia Solid Waste Management Facility. Available online: https://www.deq.virginia.gov/home/showpublisheddocument/13431/637781951047000000 (accessed on 1 February 2022).

19. Jafari, N.H.; Stark, T.D.; Thalhamer, T. Progression of elevated temperatures in municipal solid waste landfills. *J.Geotech. Geoenviron. Eng.* **2017**, *143*, 05017004.

20. Agency for Toxic Substances and Disease Registry. Landfill Gas Basics. Available online: https://www.atsdr.cdc.gov/HAC/landfill/html/ch1.html (accessed on 29 September 2023).

21. Martin, J.W.; Stark, T.D.; Thalhamer, T.; Gerbasi-Graf, G.T.; Gortner, R.E. Detection of aluminum waste reactions and waste fires. *J. Hazard. Toxic Radioact. Waste.* **2013**, *17*, 164-174, doi:10.1061/(ASCE)HZ.2153-5515.0000171.

22. U.S. Census Bureau. United States. Available online: https://www.census.gov/quickfacts/fact/table/ (accessed on 29 September 2023).

23. Virginia Department of Education. Joseph Van Pelt Elementary Profile. Available online: http://schoolquality.virginia.gov/schools/joseph-van-pelt-elementary (accessed on 5 October 2023).

24. Virginia Department of Education. Highland View Elementary Quality Profile. Available online: http://schoolquality.virginia.gov/schools/highland-view-elementary#fndtn-desktopTabs-enrollment (accessed on 5 October 2023).

25. Barbers Point Elementary School. Strive HI School Performance Report. Available online: https://arch-prod-reports-repository.s3-us-west-1.amazonaws.com/strivehi-performance/2023/251_StriveHIBarbersPointEl23.pdf (accessed on 9 November 2023).

26. Illinois State Board of Education. Illinois Report Card: Hillside Elementary School. Available online: https://irc.isbe.net/School.aspx?source=studentcharacteristics&source2=lowincome&Schoolid=060160930022001 (accessed on 9 November 2023).
